# Cross-Reactivity of Human, Wild Boar, and Farm Animal Sera from Pre- and Post-Pandemic Periods with Alpha- and Βeta-Coronaviruses (CoV), including SARS-CoV-2

**DOI:** 10.3390/v16010034

**Published:** 2023-12-23

**Authors:** Marcel Hulst, Arie Kant, José Harders-Westerveen, Markus Hoffmann, Yajing Xie, Charlotte Laheij, Jean-Luc Murk, Wim H. M. Van der Poel

**Affiliations:** 1 Department Virology & Molecular Biology, Wageningen Bioveterinary Research, 8221 RA Lelystad, The Netherlandsjose.harders@wur.nl (J.H.-W.); 2 Infection Biology Unit, German Primate Center—Leibniz Institute for Primate Research, 37077 Göttingen, Germany; mhoffmann@dpz.eu; 3 Faculty of Biology and Psychology, University Göttingen, 37073 Göttingen, Germany; 4 Institute of Food Safety and Nutrition Jiangsu Academy of Agricultural Sciences, Nanjing 210014, China; maria_xie@163.com; 5 Elisabeth-Tweesteden Hospital, 5022 GC Tilburg, The Netherlands; 6 Microvida, Elisabeth-Tweesteden Hospital, 5022 GC Tilburg, The Netherlands; j.murk@etz.nl

**Keywords:** SARS-CoV-2, α- and β-coronaviruses, cross-neutralization, pre- and post-pandemic sera, humans, farm animals, wild boars

## Abstract

Panels of pre- and post-pandemic farm animals, wild boar and human sera, including human sera able to neutralize SARS-CoV-2 in vitro, were tested in serological tests to determine their cross-reactivity with β- and α-CoV originating from farm animals. Sera were tested in neutralization assays with high ascending concentrations (up to 1 × 10^4^ TCID_50_ units/well) of β-CoV Bovine coronavirus (BCV), SARS-CoV-2, and porcine α-CoV-transmissible gastroenteritis virus (TGEV). In addition, sera were tested for immunostaining of cells infected with β-CoV porcine hemagglutinating encephalomyelitis (PHEV). Testing revealed a significantly higher percentage of BCV neutralization (78%) for sera of humans that had experienced a SARS-CoV-2 infection (SARS-CoV-2 convalescent sera) than was observed for human pre-pandemic sera (37%). Also, 46% of these human SARS-CoV-2 convalescent sera neutralized the highest concentration of BCV (5 × 10^3^ TCID_50_/well) tested, whereas only 9.6% of the pre-pandemic sera did. Largely similar percentages were observed for staining of PHEV-infected cells by these panels of human sera. Furthermore, post-pandemic sera collected from wild boars living near a densely populated area in The Netherlands also showed a higher percentage (43%) and stronger BCV neutralization than was observed for pre-pandemic sera from this area (21%) and for pre- (28%) and post-pandemic (20%) sera collected from wild boars living in a nature reserve park with limited access for the public. High percentages of BCV neutralization were observed for pre- and post-pandemic sera of cows (100%), pigs (up to 45%), sheep (36%) and rabbits (60%). However, this cross-neutralization was restricted to sera collected from specific herds or farms. TGEV was neutralized only by sera of pigs (68%) and a few wild boar sera (4.6%). None of the BCV and PHEV cross-reacting human pre-pandemic, wild boar and farm animal sera effectively neutralized SARS-CoV-2 in vitro. Preexisting antibodies in human sera effectively neutralized the animal β-CoV BCV in vitro. This cross-neutralization was boosted after humans had experienced a SARS-CoV-2 infection, indicating that SARS-CoV-2 activated a “memory” antibody response against structurally related epitopes expressed on the surface of a broad range of heterologous CoV, including β-CoV isolated from farm animals. Further research is needed to elucidate if a symptomless infection or environmental exposure to SARS-CoV-2 or another β-CoV also triggers such a “memory” antibody response in wild boars and other free-living animals.

## 1. Introduction

The spike (S) glycoprotein of coronaviruses consists of three S1–S2 heterodimers, forming a trimeric structure that protrudes from the viral envelope. The N-terminal domain of the S1 subunit (NTD) and the receptor binding domain (RBD), located at the tip of the coronavirus S protein, are crucial for the attachment of viral particles to target cells. In the context of severe acute respiratory syndrome coronavirus 2 (SARS-CoV-2), the RBD interacts with the cellular protein angiotensin converting enzyme 2 (ACE2), on the surface of cells, and this interaction is key for subsequent viral entry [1]. Furthermore, the NTD of the SARS-CoV-2 S protein may support virus attachment by binding to additional attachment factors such as 9-O-acetylated sialic acids [2]. After binding, the S1–S2 heterodimers are cleaved by host–cell proteases, a process that enables the S protein to drive the fusion of the viral and cellular membranes, resulting in the delivery of the viral genome into the cytoplasm for genome replication and translation. Notably, the NTD and RBD constitute crucial immunogenic regions [3]. Consequently, neutralization of SARS-CoV-2 in vivo by antibodies induced by infection or vaccination mainly occurs by binding of antibodies to epitopes located within the NTD and RBD [3,4], and references herein. The worldwide scale of the COVID-19 pandemic and the massive vaccination campaigns of humans have imposed huge immunological pressure on these domains. This pressure resulted in the evolution of several SARS-CoV-2 variants, including variants of concern (VOCs), bearing mutations that weakened or abrogated the binding of neutralizing antibodies [4,5] and references herein. As a result, mRNA-based COVID-19 vaccines were rapidly adapted to match globally dominating SARS-CoV-2 variants that show a high level of neutralization resistance against antibodies elicited by vaccination with “classical” SARS-CoV-2 vaccines, which are formulated based on viruses circulating at the beginning of the pandemic [5]. Since none of the vaccines provide total protection against SARS-CoV-2 infection and novel antibody-evasive SARS-CoV-2 lineages keep emerging [6], booster vaccinations with updated vaccines are required to support protection, particularly in vulnerable populations, such as elderly and immunocompromised people. The development of vaccines that offer prophylactic protection against contemporary as well as newly emerging SARS-CoV-2 lineages and, ideally, also against possible future zoonotic β-coronaviruses (β-CoV) that may spill over from wild animals, could reduce the need for booster vaccination and may prevent repetition of an uncontrollable pandemic phase, as seen in the case of SARS-CoV-2 in 2020.

Several studies reported that IgG antibodies directed against epitopes of the “common cold” β-CoV (e.g., human coronavirus (HCoV)-OC43 and HCoV-HKU1) in human sera cross-reacted with SARS-CoV-2 nucleoprotein (NP) and S protein antigens [7,8]. Significantly higher concentrations of these cross-reacting antibodies, or antibodies with a higher binding affinity, were present in sera of humans that experienced a SARS-CoV-2 infection compared to sera of humans who were not exposed to SARS-CoV-2 (including pre-pandemic sera). Thus, SARS-CoV-2 infection induced a memory immune response, presumably initiated by CD4+ T cells recognizing similar or structurally related epitopes on the viral surface of SARS-CoV-2 and common cold β-CoV [9,10,11]. It has been suggested that activation of this memory response by SARS-CoV-2 could be one of the factors preventing a severe course of COVID-19 disease in specific individuals [11]. Recently, it was also shown that circulating antibodies in SARS-CoV-2-convalescent individuals reacted with conserved linear epitopes encoded in the S2 subunit of the spike protein of several coronaviruses [12]. Monoclonal antibodies targeting these so-called “cold-spot epitopes” in S2 were able to protect human-ACE2 sensibilized mice against the development of severe disease upon SARS-CoV-2 challenge [12]. This suggested that the construction of vaccines providing partial or complete protection against a broad collection of β-CoV, including SARS-CoV-2 VOC’s and coronaviruses that may cause future spillovers, could be feasible.

In the spring of 2020, SARS-CoV-2 spilled over from humans to farmed minks. SARS-CoV-2 rapidly spread among mink farms in The Netherlands and spilled back from minks to humans [13]. Spill-over and spill-back events were also reported for free-ranging white-tailed deer in the USA [14]. Although various companion animals (including cats and dogs), wildlife and livestock species are susceptible to SARS-CoV-2 infection, most of the reported natural cases remained asymptomatic or displayed mild respiratory signs, and no massive spread among these animals occurred [15] and references herein. To our knowledge, only a few spill-over and spill-back events from hamster and cat pets to humans were reported [16,17]. In contrast, under laboratory conditions, cats, hamsters and ferrets were highly susceptible to infection with human SARS-CoV-2 isolates, showing typical COVID-19 clinical signs, and they transmitted the virus to naïve contact animals [18,19]. No replication of SARS-CoV-2 was reported for poultry, whereas replication in a few pigs, cattle and sheep was only reported after experimental infection with SARS-CoV-2 [19]. Also, no severe COVID-19 clinical signs were observed in these experimental studies in pigs [20], cattle and sheep [18,19], and shedding and transmission of SARS-CoV-2 to naïve contact animals were not detected. Moreover, for pigs, sheep and cattle in production sites, farm-scale outbreaks or even sporadic infections of SARS-CoV-2 were not reported [18,19]. This suggests that efficient replication of currently circulating SARS-CoV-2 variants is not supported by these farm animals. Nevertheless, the continuing occurrence of new SARS-CoV-2 variants, which may have acquired mutations supporting more efficient replication in these economically important animals, is a serious threat and demands ongoing surveillance.

In addition to host-specific factors, like the structure of the ACE2 receptor and the fitness of the immune system, antibodies elicited by past infections with CoV that cross-react with SARS-CoV-2 could also contribute to the observed (partial) resistance of farm animals to SARS-CoV-2 infection (as suggested for common-cold-associated CoV in humans). In this study, we made an inventory of β-CoV cross-reacting antibodies in animal and human sera. Through neutralization tests, enzyme-linked immunosorbent assay (ELISA) and immune staining of infected cells, we tested panels of pre- and post-pandemic sera from pigs, cattle (dairy cows), goats, sheep, rabbits, wild boars and humans, including SARS-CoV-2-convalescent sera (from PCR-positive individuals), for the presence of antibodies that cross-react with heterologous CoV, i.e., the β- CoV SARS-CoV-2, bovine coronavirus (BCV), porcine hemagglutinating encephalomyelitis virus (PHEV) and the α-CoV-transmissible gastroenteritis virus (TGEV). In Central Europe, BCV and PHEV are endemic in cattle and pig populations, respectively [21,22]. The protein sequence of the spike proteins of BCV and PHEV is 92% and 81% identical, respectively, to that of the human common cold coronavirus HCoV-OC43 [23]. TGEV is the ancestor virus of porcine respiratory coronavirus (PRCV) [24]. Compared to its enteric ancestor, PRCV switched to a respiratory virus due to a few nucleotide mutations and deletions in the genomic regions coding for the S protein and ORF3 [24]. Since PRCV became endemic, pigs developed immunity to both PRCV and TGEV [22].

To avoid false-positive results due to nonspecific binding of serum antibodies, for testing of the abovementioned heterologous serum panels, we developed robust neutralization tests for SARS-CoV-2, TGEV and BCV. Sera were tested at a relatively high (mostly fixed) starting dilution of 1:50 and incubated with high ascending concentrations of target virus. Developed neutralization tests were validated using sera of naïve animals and animals experimentally infected with α- and β-CoV. In addition, we raised antibodies in rabbits against a stretch of 13 aa residues, corresponding with the N-terminal part of the CoV fusion peptide (FP), positioned downstream of the S2′ cleavage site (recently also identified as FP “cold spot epitope” involved in neutralization of SARS-CoV-2 [12]), and we tested these antibodies on their ability to stain cells infected with SARS-CoV-2 and several α- and β-CoV originating from farm animals.

## 2. Materials and Methods

### 2.1. Cells and Viruses

Vero cells (CCL-81), Vero-E6 (CRL-1586™), EBTR (NBL-4, CCL-44™) and DF-1 chicken fibroblasts (CRL-3586) were obtained from ATCC^®^. Swine kidney cells SK6 [25] were obtained from our own collection of cells. RK13 cells CCL-37™ were kindly provided by Dr. Herman Egberink of Utrecht University, Utrecht, The Netherlands. All ATCC^®^ cell lines were maintained in the culture medium recommended by the ATCC^®^ supplemented with 10% *v*/*v* fetal bovine serum (FBS) and 1% *v*/*v* antibiotics (Antibiotic-Antimycotic, 100× Gibco™, Thermo Fisher Scientific, Bleiswijk, The Netherlands). SK6 cells were maintained in Dulbecco’s Modified Eagle’s Medium supplemented with 10% *v*/*v* FBS and 1% *v*/*v* antibiotics. The FBS was free of antibodies directed against bovine viral diarrhea virus. Porcine epidemic diarrhea virus (PEDV) strain CV777, bovine respiratory syncytial virus (BRSV) strain RB94, infectious bronchitis virus (IBV) strain Baudette and TGEV strain Purdue were obtained from our own collection of viruses. The rabbit fibroma virus (RFV-VR-112™) was obtained from ATCC^®^. An enteric bovine coronavirus (BCV) strain was isolated from cattle in Sweden and was kindly provided by the Swedish University of Agricultural Sciences (SVA) in 2007 [26]. The PHEV strain VW572 was kindly provided by Prof. Hans Nauwynck of the University of Ghent, Belgium [23]. Isolation of the WBVR SARS-CoV-2 strain “human/NLD/Lelystad/2020” (GenBank accession number MZ144583) was described by Gerhards et al. [27]. Compared to the nucleotide sequence of the first SARS-CoV-2 isolate from a human in Wuhan, China (Wuhan-Hu-1 strain, acc. number NC_045512.2), this WBVR isolate contains three nucleotide differences, resulting in one amino acid (aa) exchange in the spike protein at position 614 (D614G). 

### 2.2. Pre-Treatment of Sera

All sera of naive animals, experimentally infected animals, farm animals, humans and wild boar were incubated for 30 min at 56 °C to inactivate complement factors. In a part of the sera collected from hunted wild boars, a sediment appeared after incubation at 56 °C. After centrifugation of these sera for 5 min at 500× *g,* the supernatant was isolated from these sera and used in serological tests.

### 2.3. Immune Staining of Infected Cells 

Confluent monolayers of cells grown in 2 cm^2^ tissue culture wells (M24 plates) were infected with an appropriate dilution of virus stock and grown until the cytopathogenic effect was observed microscopically for about 20–50% of the cells in a well (depending on the combination of virus and cell line, cells were grown 2 to 7 days at 37 °C and 5% CO_2_). Wells were washed twice with phosphate-buffered saline (PBS) and fixed using 4% *w*/*v* paraformaldehyde in PBS for 10 min at RT. After discarding the paraformaldehyde, wells were incubated with 100% methanol for 5 min, whereafter plates were washed twice with PBS and incubated with PBS containing 1% *v*/*v* Triton X100 for 10 min at RT. Plates were washed twice with PBS and pre-incubated for 30 min with PBS containing 5% *v*/*v* horse serum (PBS-H). The PBS-H was discarded, and wells were incubated with appropriate dilutions of antibody preparations or test sera in PBS-H for 1 h at 37 °C. Plates were washed twice with PBS and incubated for 1 h at 37 °C with an appropriate dilution of horse-radish peroxidase (HRP)-conjugated secondary antibodies, specifically directed against Ig (H + L) chains of the species serum tested. After washing the plates three times with PBS, infected cells were stained using 3-amino-9-ethylcarbazole (AEC) as a substrate. After 2 h of staining, the AEC substrate solution was discarded; plates were then washed with demineralized water and air-dried. Dried plates were scanned using AID vSpot imaging apparatus (AID Autoimmun Diagnostika GmbH, Strassberg, Germany) to count the number of infected foci in each well. In Supplementary Figure S3, the strength of PHEV staining by a test serum is described, assigned to one of the three categories, (i) negative ([−]: no staining or background staining), (ii) positive ([+]: cytoplasmic staining at a serum dilution of 1:250), or (iii) strong positive ([++] cytoplasmic staining at a serum dilution of 1:750). In Supplementary Table S2, specifications of the HRP-conjugated secondary antibodies used to detect bound Igs of different animals and humans are provided.

### 2.4. Neutralization Assays

#### 2.4.1. M96 SARS-CoV-2 VNT

Standard SARS-CoV-2 neutralization assay (VNT) in M96 plates was performed as described by Gerhards et al. [27], with minor modifications. Briefly, sera were tested in serial dilutions in triplicate in three columns of eight wells in M96 plates. Four-fold or three-fold serial dilutions were prepared in culture medium using a starting dilution of five- or ten-fold, respectively. About 50 µL of medium containing 50 TCID_50_ of SARS-CoV-2 and 50 µL of medium containing 15,000 Vero-E6 cells were added, and after 4–5 days of growth, SARS-CoV-2-infected cells were detected by immunostaining, as described above. The percentage of infected cells in each well was determined using the AID vSpot imaging apparatus. Log2 values of the highest dilutions of serum-neutralizing infection in a well for ≥50% were used in the formula “= POWER(2,(log2 dilutions-column 1 + log2 dilutions-column 2 + log2 dilutions-column 3)/3)” to calculate the VNT titer.

#### 2.4.2. BCV, SARS-CoV-2 and TGEV M24 Neutralization Assays

Virus stocks of BCV, SARS-CoV-2 or TGEV with known titer were diluted in serum-free medium (EX-CELL^®^ MDCK Serum-Free Medium for MDCK Cells, Sigma Aldrich, Zwijndrecht, The Netherlands) to give three (TGEV) or four (SARS-CoV-2 and BCV) ascending concentrations of virus (specified below). For each test serum, 220 µL of each virus dilution was transferred to three or four empty 2 cm^2^ tissue culture wells (in a clean “dummy” M24 plate). Test sera were diluted 1:25 in serum-free medium, and to each of the three or four wells containing a different amount of virus, 220 µL of this serum dilution was added and mixed, making the final serum dilution that was tested 1:50. M24 plates with 440 µL serum–virus mixtures were pre-incubated for 30 min at 37 °C and 5% CO_2_. The growth medium of M24 plates with nearly confluent monolayers of EBTR (BCV), Vero-E6 (SARS-CoV-2) or SK6 cells (TGEV) was discarded, and wells were washed once with PBS and once with serum-free medium. After discarding the serum-free wash medium, 350 µL of the serum–virus mixtures was transferred from the pre-incubated “dummy” plate to the same well positions of the M24 plate with cells and incubated for an infection period of 90 min at 37 °C and 5% CO_2_. After the infection period, 650 µL of the appropriate growth medium was added to each well, and cells were grown for 2 days (EBTR-BCV), 4 days (Vero E6-SARS-CoV-2) or 6–7 days (SK6-TGEV, see below) at 37 °C and 5% CO_2_. On each day of testing, a virus-specific neutralizing serum (positive control), a non-neutralizing serum (negative control) and three or four wells without serum (i.e., only containing the three or four ascending amounts of virus) were tested along with panels of field sera. The four ascending amounts of virus (for TGEV, only three) present during pre-incubation and infection periods were 10, 100, 1000 and 10,000 TCID_50_/well of SARS-CoV-2, 5, 50, 500 and 5000 TCID_50_/well of BCV and 100, 1000 and 10,000 TCID_50_/well of TGEV. All field sera were tested with the three highest amounts of SARS-CoV-2 and BCV per well. Note that for validation purposes, serum dilutions of 1:100 and 1:200 were also tested in BCV and SARS-CoV-2 M24 neutralization assays in combination with the four abovementioned amounts of virus per well (checkboard titration layout).

#### 2.4.3. Determination of the Neutralization Index (NI)

BCV- and SARS-CoV-2-infected cells in wells were detected by immune staining, as described above. A 1:300 dilution of the αS2′-IgG preparation was used as primary antibody and a 1:300 dilution of Goat-anti-Rabbit (H + L)-HRP as conjugate. After 2 h of staining, the AEC substrate solution was discarded; plates were washed with demineralized water and air-dried. Dried plates were scanned using an AID vSpot imaging apparatus to count the number of infected foci in each well (blue numbers displayed in the left-lower corner of the images of each well). The number of infected foci in wells incubated with test sera and wells incubated with a negative serum, both incubated with the same amount of virus, was used to calculate the percentage of infected cells in wells with test serum using the formula (# foci test serum/average # foci in 3 or 4 wells with no serum) × 100%. The BCV-NI or SARS-CoV-NI of a test serum was defined as the highest amount of virus neutralized for ≥90% by a 1:50 dilution of serum. After 2 days of growth, parts of the monolayers of SK6 cells were already disconnected from the bottom of the wells due to the cytopathogenic effect (CPE) induced by TGEV. This loss of cells made counting the TGEV-infected foci after fixation and immune staining inaccurate. Therefore, plates were incubated for another 4 to 5 days. After this extended period, the medium of wells in which no TGEV infection had occurred colored yellow (due to overgrowth of the SK6 cells), and the medium in wells in which 100% CPE was induced stayed red (see Supplementary Figure S2). The TGEV-NI of a test serum was defined as the highest amount of virus neutralized for 100% by a serum dilution of 1:50.

#### 2.4.4. Pseudovirus Neutralization Assay

The pseudovirus neutralization assay was conducted at the Infection Biology Unit of the German Primate Center in Göttingen, Germany, according to a previously published protocol [28]. Pseudoviruses bearing VSV-G, wildtype or mutant SARS-CoV-2-S [29] were pre-incubated for 30 min at 37 °C with serial dilutions of antibody and subsequently inoculated onto Vero cells. Pseudoviruses incubated with medium without antibody served as control. After an incubation period of 16–18 h, pseudovirus infection was analyzed through the measurement of luciferase activity in cell lysates. For this, cells were lysed in PBS containing 0.5% triton X-100 (Carl Roth, Karlsruhe, Germany) for 30 min at room temperature, before lysates were transferred into white 96-well plates (Greiner Bio-One, Kremsmünster, Austria). Next, luciferase substrate (Beetle-Juice, PJK, Kleinblittersdorf, Germany) was added, and luminescence was recorded using a Hidex Sense Microplate Reader (Hidex, Turku, Finland). Neutralization efficiency was calculated based on the relative inhibition of pseudovirus entry, using signals obtained from pseudovirus particles incubated in the absence of an antibody as reference (=0% inhibition).

### 2.5. SARS-CoV-2 NP and S ELISA 

Sera were tested in indirect ELISAs for the presence of antibodies directed against the nucleocapsid protein (NP) and the complete spike protein (S1–S2) of SARS-CoV-2, as described recently [30]. Briefly, recombinant NP and S1–S2 proteins produced in insect cells (ECD-His tagged; both from Sino biological, Eschborn, Germany) were coated overnight in 50 mM NaHCO_3_ (pH 9.6) at 4 °C at a concentration of 50 ng/100 µL in M96 plates (100 µL/well). After each incubation step, plates were washed three times with PBS-0.05% *v*/*v* Tween 20 using a washing machine. After a blocking step for 1 h at 37 °C with 100 µL/well StabilBlock^TM^ solution (SURMODICS: cat.nr ST-1-1000, Eden Prairie, MN, USA), plates were incubated for 1 h at 37 °C with serial dilutions of pre-treated sera in PBS-0.05% *v*/*v* Tween 20 containing 5% *v*/*v* horse serum (PBS-Tw-HS). Subsequently, plates were incubated for 1 h at 37 °C with an appropriate dilution in PBS-Tw-HS of horseradish peroxidase (HRP)-conjugated secondary antibodies specifically directed against Ig(H + L) chains of the species sera under study (for details, see Supplementary Table S2 and reference [31]). Plates were colored at RT using 100 µL/well ready-to-use 5′-Tetramethylbenzidine (TMB) substrate solution (1-StepTM Ultra TMB-ELISA Substrate Solution, Thermo Fisher Scientific, Bleiswijk, The Netherlands). The color reaction was stopped after 5 min by adding 100 µL/well 0.5 M H_2_SO_4_, and absorption was measured at 450 nM using a Molecular DevicesSpectraMax^®^ ABS Plus plate reader (San José, CA, USA). The “Absorbance summation” factor (summation of the absorption values of all tested dilutions: AS [32]) was calculated. Because different species-specific HRP conjugates were used to detect bound immunoglobulins, all displaying a different level of background staining, sera were considered as a positive (cross)-reactor in case an AS ≥ 3 was measured. An AS of 3 corresponded with about 3-times the AS values measured for sera collected from naïve piglets, rabbits and calves.

### 2.6. Farm Animal and Human Sera Used for Immune Staining, Neutralization Tests and ELISAs 

In Supplementary Table S1, details and references about the origins of the pre- and post-pandemic human and animal sera used in this study are described. Sera were collected from hunted wild boars in the Veluwe and Peel regions in The Netherlands in 2018 (pre-pandemic sera) and during the pandemic from January 2021 to March 2022 (post-pandemic sera). For locations, see the map in Supplementary Figure S5. A panel of pre-pandemic human sera collected in 2018 at the ETZ hospital in Tilburg in the Netherlands (*n* = 52) consisted of 30 sera of random patients (H-ETZ-P) and of 22 sera from farmers of pig farms located in the province of North Brabant (H-ETZ-F). A panel of sera collected early after the start of the pandemic in The Netherlands (March until April 2020) consisted of 72 sera of healthcare workers and intensive care patients of the ETZ hospital, which all tested positive for SARS-CoV-2 by PCR (hereafter denoted as SARS-CoV-2-convalescent sera). For these 72 sera, the SARS-CoV-2 VNT titer was determined in a “standard neutralization” test at the “National Institute for Public Health and the Environment” in The Netherlands (RIVM) as part of a serological validation program. In addition, 11 sera of WBVR lab workers (H-WBVR), from which 6 tested positive for SARS-CoV-2 by PCR [33] and 5 negative, were also included in this study. The VNT titer for these 11 sera was determined at WBVR in a “standard” M96 neutralization test for SARS-CoV-2 [27]. In Supplementary Table S1, details of all sera collected from laboratory animals experimentally infected with α- and β-CoVs and sera from naive farm animals available at our institute (CD-CD calfs and piglets, SPF sheep or rabbits from a “high health” farm) are described. Due to the limited volumes available, not all sera were tested in all the serological tests conducted in this study.

### 2.7. Statistical Analysis

Chi-square tests of independence were conducted at an alpha of 5% (*p*-value < 0.05) comparing the number of sera per BCV-NI or PHEV staining category between panels of pre- and post-pandemic sera. Tests were performed separately for panels of human sera and wild boar sera. In Supplementary Figure S4A, contingency tables and Chi-square values (*X*^2^) are provided.

## 3. Results

### 3.1. Rabbit Antibodies Raised against S2 Peptide React with Heterologous α- and β-CoVs In Vitro but Are Non-Neutralizing

**Figure 1:**
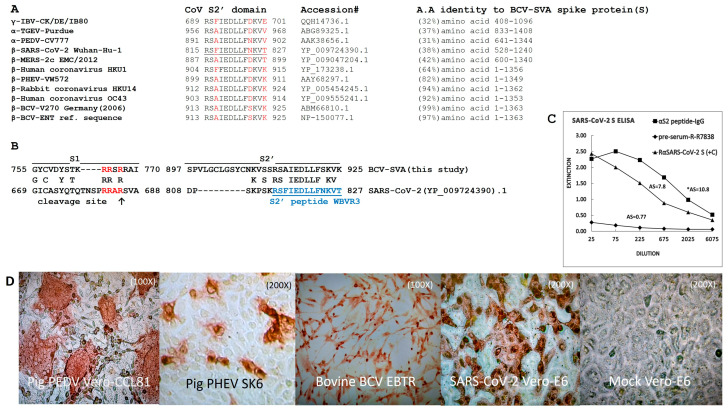
Characterization of a rabbit serum raised against S2′peptide of CoV. (**A**) Amino acid (aa) sequence of the S2′ peptide domain (underlined for SARS-CoV-2) in the spike proteins of human and animal CoV. Percent identity of aa sequences (Accession#) of the spike proteins of selected CoV to that of BCV strain SVA (used in this study) are indicated. The protein sequence downstream of the S2′ cleavage site has high sequence identity (%) among diverse CoV. (**B**) Protein sequences of the S1/S2 region and S2′ cleavage site (↑) in the spike proteins of SARS-CoV-2 and BCV-SVA. The sequence of the S2 peptide used for immunization of rabbits is depicted in blue and the S1–S2 cleavage site in the spike protein is indicated in red. (**C**) Reactivity of the αS2′-IgG antibody fraction, purified from bleeds of rabbits, in an indirect ELISA coated with recombinant spike protein of SARS-CoV-2 produced in insect cells. RαSARS-CoV-2 S (+C): rabbit positive control serum raised against the spike protein of SARS-CoV-2 ([27]), pre-serum-R-R7838: serum collected from the rabbit before it was immunized with the S2’ peptide WBVR3. AS; “Absorbance summation” factor ([32]: summation of the absorption values of all tested dilutions). (**D**) Indirect immune staining of cells infected with SARS-CoV-2 and α- and β-CoV isolated from farm animals using the αS2′-IgG antibody fraction.

A peptide of 13 aa residues, located at the N-terminus of S2, was selected (Figure 1A,B), corresponding with aa residues 815–827 in the spike protein of the SARS-CoV-2 Wuhan-Hu-1 strain (hereafter denoted as S2′ peptide), and was synthesized and used for immunization of rabbits. Peptide synthesis, coupling to Keyhole limpet hemocyanin (KLH), immunisation of rabbits with the KLH peptide (three successive injections) and affinity-chromatography purification of “αS2′-IgG” antibodies from bleeds after three immunization, was performed using a commercial service (GenScript Biotech B.V., Rijswijk, The Netherlands). The reactivity of the purified αS2′-IgG antibody fraction was tested by immune staining of cultured cells infected with γ-CoV avian infectious bronchitis virus (IBV), α-CoV PEDV and TGEV, and β-CoV PHEV, BCV, and SARS-CoV-2. Cytoplasmic staining of virus-infected cells was observed for all three tested β-CoV, i.e., PHEV, BCV and SARS-CoV-2, and for both the α-CoV PEDV and TGEV (Figure 1D, shown for PEDV). No staining was observed for γ-CoV IBV, rabbit fibroma virus and bovine respiratory syncytial virus (BRSV)-infected cells and all types of mock-infected cells used in this study (only shown for mock-infected Vero-E6 cells in Figure 1D). The specificity of the αS2′-IgG antibody fraction for the spike protein of CoV was confirmed in an indirect ELISA (see Material and Methods) using recombinant spike (S1–S2) or NP proteins of SARS-CoV-2 produced in insect cells as coating (Sino biological, Eschborn, Germany). The αS2′-IgG antibodies effectively bound to the coated recombinant spike (S1–S2) protein of SARS-CoV-2, whereas pre-serum collected from the rabbit before immunisation showed no binding (Figure 1C). The αS2′-IgG antibodies did not react with recombinant NP of SARS-CoV-2 when used as coating. To test whether the αS2′-IgG antibody fraction exerts neutralising activity against SARS-CoV-2, we employed a pseudovirus neutralization assay based on vesicular stomatitis virus (VSV), harbouring either the VSV glycoprotein (VSV-G, control) or the SARS-CoV-2 S protein (SARS-2-S) [29]. While the αS2′-IgG antibody did not inhibit Vero cell entry of pseudoviruses bearing VSV-G, as expected, it was also unable to block the entry of pseudoviruses bearing SARS-2-S, not even at high concentrations. Furthermore, it did not make any difference if SARS-2-S mutants were tested that possess modified S1/S2 cleavage sites, which abolish or improve S protein processing by furin (Supplementary Figure S1). In addition, no neutralization of SARS-CoV-2 and BCV infection in Vero-E6 and EBTR cells, respectively, was observed for the αS2′-IgG antibody fraction.

### 3.2. Rabbit and Human Sera Stain Cells Infected with Heterologous β-CoV, including SARS-CoV-2

**Figure 2:**
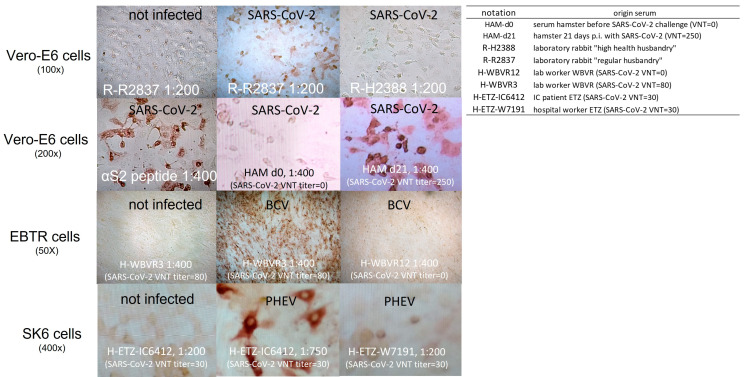
Immune staining of β-CoV infected cells with animal and human sera. Details about the origin of the sera are provided in Supplementary Table S1. Dilutions of serum used as primary antibody to stain infected cells are specified directly after the annotations of the sera. SARS-CoV-2 VNT titers measured for hamster and human sera in the standard M96-SARS-CoV-2 VNT are indicated between brackets.

Immune staining of β-CoV-infected cells revealed that particular sera from five- to seven-week-old laboratory rabbits (pre-immunization sera: collected before immunized), held under a regular husbandry regime, stained Vero-E6 cells infected with SARS-CoV-2 (Figure 2: shown for serum R-R-2837) and also cells infected with BCV and PHEV. Compared to serum collected from a hamster 21 days after experimental infection with SARS-CoV-2 (HAM d21), staining was less intense for these rabbit sera. No staining was observed for sera collected from four rabbits of the same age raised in a “high health” husbandry (Figure 2: shown for serum R-H-2388). Human SARS-CoV-2-convalescent sera with known SARS-CoV-2 VNT titer, as well as some sera of humans, tested negative for SARS-CoV-2 with PCR (without neutralizing activity for SARS-CoV-2, VNT = 0) and were also able to stain cells infected with BCV and PHEV. Intense cytoplasmic staining of BCV- and PHEV-infected cells was observed for several of these human sera (Figure 2: only shown for SARS-CoV-2 convalescent sera H-WBVR-3 and H-ETZ-IC6412). However, not all of the tested SARS-CoV-2 VNT-positive sera, collected from individuals, stained PHEV- or BCV-infected cells (e.g., H-ETZ-W7191 Figure 2).

### 3.3. Human Pre-Pandemic and SARS-CoV-2-Convalescent Sera Neutralize the Heterologous β-CoV BCV In Vitro but Not the α-CoV TGEV

**Figure 3:**
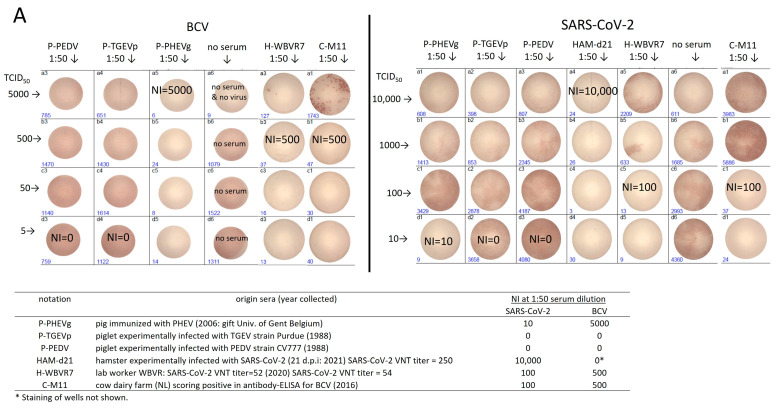

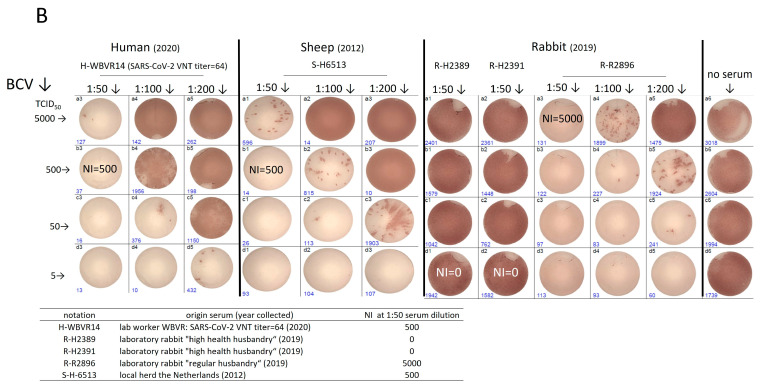
Neutralization of coronavirus infection by homologue and heterologous sera. (**A**) BCV and SARS-CoV-2 neutralization by sera of experimentally infected animals. SARS-CoV-2 VNT titers measured for the human H-WBVR# and hamster sera in the standard M96-SARS-CoV-2 VNT are indicated between brackets and in the inserted table of Figure 3A. (**B**) Serum and virus concentration dependent neutralization of BCV. Neutralization index (NI): TCID_50_/ per 2 cm^2^ well of BCV or SARS-CoV-2 for ≥90% neutralized by an 1:50 dilution of serum. The number of infected foci in wells are indicated in blue in the lower left corner of each well. Details about the origin of the sera are provided in Supplementary Table S1.

To validate the specificity of M24 neutralization tests for BCV, SARS-CoV-2 and TGEV, a set of experimental sera was tested. The BCV strain used for the BCV neutralization test was an enteric strain isolated from cattle in Sweden in 2003 (in this study named “BCV-SVA”). The strain was sequenced by NGS after two passages in EBTR cells to determine the protein sequence of the S and NP proteins. The protein sequence of the BCV-SVA S protein was, for 97%, identical to the sequence of the S protein of the BCV-ENT reference strain posted in the NCBI database (acc. number NP-150077.1) and for 92% to the S protein of the human common cold β-CoV HCoV-OC43 (YP_009555241.1). The protein sequence of the BCV-SVA NP protein was, for 98%, identical to NP of BCV-ENT (NP_150083.1) and for 97% to HCoV-OC43 NP (YP_009555245.1). In Figure 3, the results of validation of the M24 neutralization assays for BCV and SARS-CoV-2 are depicted, and for TGEV in Supplementary Figure S2. The determined neutralization index (NI) at a serum dilution of 1:50 is provided in the table inserted in Figure 3. None of the sera collected from naïve animals (shown for two sera of rabbits held in a high-health farm [R-H#] in Figure 3B) and from PEDV- and TGEV-seroconverted piglets (Figure 3A) showed cross-neutralizing activity towards SARS-CoV-2 and BCV in the M24 neutralization test, not even in the case of low concentrations of virus applied (5 or 10 TCID_50_/well). In the M24 assay, serum of a naïve laboratory hamster experimentally infected with SARS-CoV-2 (Ham-d21: M96 VNT = 250 Figure 3A) [27] and a SARS-CoV-2-convalescent human serum (H-WBVR7 M96 VNT = 54) neutralized SARS-CoV-2 with an NI of 10,000 and 100, respectively. The strength of cross-neutralization of BCV by another SARS-CoV-2-convalescent human serum (H-WBVR14 M96 VNT = 64), a pre-pandemic sheep serum (Figure 3B, S-H6513) and a pre-pandemic serum of a rabbit held on a regular farm (Figure 3B, R-R2896) was dependent of the concentration of BCV and serum dilution. Only 1 out of 16 pre-pandemic cow sera that effectively neutralized BCV (see Section 3.4) showed cross-neutralizing activity towards SARS-CoV-2 in the M24 assay (shown for C-M11 NI = 100, Figure 3A). Although the sera of piglets immunized with the β-CoV PHEV (P-PHEVm) or experimentally infected with PHEV (P-PHEVg) effectively cross-neutralized BCV, we observed only weak (P-PHEVg NI = 10, Figure 3A) or no (P-PHEVm) neutralizing activity towards SARS-CoV-2 in the M24 neutralization assay (only shown for P-PHEVg). 

To validate whether cross-neutralization by human sera and heterologous animal sera was restricted to the β-CoV BCV, human, cow and pig sera able to (cross)-neutralize SARS-CoV-2 and/or BCV were tested at a dilution of 1:50 in the M24 neutralization test for the α-CoV TGEV (see Supplementary Figure S2). Homologous sera of piglets experimentally infected with the α-CoV TGEV and/or PRCV (P-PRCTGEVm and P-TGEVp) effectively neutralized TGEV. SARS-CoV-2-convalescent human sera (H-WBVR#) and sera of piglets experimentally infected α-CoV PEDV (strain CV777) or immunized with PHEV (PHEVg) showed no neutralizing activity against TGEV. From the tested heterologous sera, only the serum of the pig experimentally infected with the β-CoV PHEV (P-PHEVg) was able to neutralize TGEV (NI = 1000).

### 3.4. Testing of Panels of Pre- and Post-Pandemic Human and Animal Sera for Cross-Reactivity with CoV

Panels of pre- and post-pandemic sera from humans, wild boar and farm animals were tested in the validated BCV and TGEV M24 neutralization tests, as well as for their ability to stain SK6 cells infected with PHEV. In the Section 2.3 and Supplementary Figure S3, the strength of PHEV staining by a serum sample is described, and it was assigned to the categories (i) negative [−], (ii) positive [(1:250)+], or (iii) strong positive [(1:750)++]. In Table 1, the results of the BCV and TGEV neutralization tests and PHEV staining are presented. 

No cross-neutralization of BCV was observed for pre-pandemic goat sera collected from two different farms. Cross-neutralization of BCV was observed only for pre-pandemic sheep sera collected from one of the three herds. None of the BCV-neutralizing sera of sheep, cows and rabbits showed any cross-neutralizing activity for TGEV. Almost half of 42 pre-pandemic sera of finishing pigs (randomly picked from a collection of 1000 sera collected at a slaughterhouse in 2007; P-F panel) effectively neutralized BCV and TGEV. Most of these BCV-neutralizing sera also stained PHEV-infected cells. A comparable BCV and TGEV neutralization and PHEV-staining pattern was observed for post-pandemic sera of pigs from two fattening farms. In contrast, pre- and post-pandemic pig sera collected from two other farms, P-Exp and farm P-S/P-P (sows and piglets from the same farm), respectively, showed little or no neutralizing activity for BCV, whereas most of these sera effectively neutralized TGEV. 

**Figure 4:**
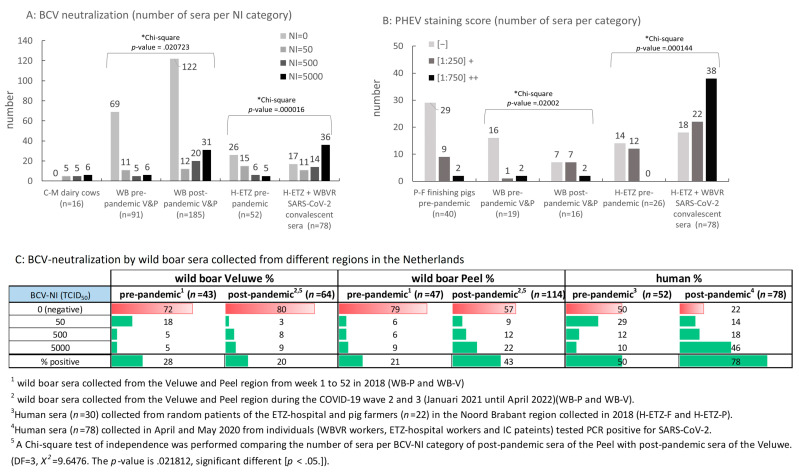
BCV-neutralization and PHEV-staining by human and wild boar sera. (**A**) Number of pre-pandemic and post-pandemic wild boar sera (WB; V&P, Peel and Veluwe regions displayed together, see Supplementary Figure S5 for details) and human (H) pre- and post-pandemic sera (SARS-CoV-2 convalescent) scoring a BCV-NI of 50, 500, or 5000 TCID_50_/well and (**B**) stained PHEV infected cells at a serum dilution of 1:250 [+] or 1:750 [++], or did not stain PHEV infected cells [−] at a dilution of 1:250. C-M: pre-pandemic sera of dairy cows containing antibodies directed against BCV. P-F: pre-pandemic sera of randomly selected finishing pigs. *Chi-square test of independence *p*-values (see Supplementary Figure S4A for details). (**C**) Percentages of pre- and post-pandemic (SARS-CoV-2 convalescent) human and wild boar sera (separately displayed for the Veluwe and Peel region) scoring a BCV-NI of 50, 500, or 5000 TCID_50_/well. Green bars, “% positive” = total percentage of sera able to neutralize BCV. Red bars, “0 (negative)”: percentage of sera not able to neutralize 50 TCID_50_/well BCV at a serum dilution of 1:50.

None of the pre-pandemic and post-pandemic SARS-CoV-2-convalescent human sera showed any neutralizing activity towards TGEV. Compared to pre-pandemic human sera of random patients (H-ETZ-P), a significantly higher percentage of SARS-CoV-2-convalescent sera neutralized BCV infection (78% post- versus 37% pre-pandemic). Remarkably, a higher percentage of BCV-neutralizing sera was also observed for pre-pandemic sera collected from pig farmers (68% for the H-ETZ-F panel). For all three pre- and post-pandemic panels of human sera, a similar pattern of cross-reactivity was observed in the PHEV staining test as in the BCV-neutralization test. Furthermore, the percentage of sera able to cross-neutralize a high concentration of BCV, i.e., 5000 TCID_50_/well, was significantly higher for SARS-CoV-2-convalescent human sera than for pre-pandemic human sera (Figure 4A: pre-pandemic H-ETZ-P plus H-ETZ-F panels displayed together). Moreover, for a majority of the post-pandemic SARS-CoV-2-convalescent human sera, a higher dilution (lower concentration) of serum (Figure 4B: [1:750]++ category) achieved positive staining of PHEV-infected cells, whereas for pre-pandemic human sera, positive staining was observed only at a dilution of 1:250. For the panel of convalescent human sera with BCV-neutralizing activity (*n* = 60), no correlation was found between titers determined in the standard M96 SARS-CoV-2 VNT test and BCV-NI scores or serum dilutions that scored positive PHEV staining (see Supplementary Figure S4B).

A high percentage of pre- and post-pandemic pig sera from all sampled farms neutralized TGEV infection. Despite BCV neutralization being observed for a considerable number of pre- and post-pandemic sera from finishing/fattening pigs (PF and PFA panels), only a few sera originating from two particular farms (P-S and P-P panels from the same farm, and panel P-Exp) weakly neutralized BCV (see Table 1). Obviously, past infection(s) with an α-CoV occurred in all pig farms tested, while past infection(s) with a β-CoV may only have occurred in individual farms, or infection(s) with a β-CoV in the farms induced an antibody response not strong enough to obtain neutralizing activity against heterologous BCV. These farm-specific profiles observed for pigs prompted us to test sera of a non-cloistered population of *Sus scrofa*. Large panels of pre- and post-pandemic sera collected from hunted wild boars were tested for cross-neutralization in BCV and TGEV tests. Wild boars were hunted in two distantly located regions, separated by the three main rivers flowing through The Netherlands, i.e., the Veluwe region and the Peel region (for the location of these regions, see map in Supplementary Figure S5). The Veluwe is a nature reserve park, mainly consisting of forest areas that are partly, on a temporary basis, accessible to the public. The Peel region is located in the southeast of The Netherlands and is a mix of free-accessible forest and agricultural areas in the vicinity of cities and villages. In contrast to pigs, only a few pre- and post-pandemic sera of wild boars showed neutralizing activity for TGEV (Table 1). Thirty-four percent of the post-pandemic wild boar sera (Veluwe plus Peel region; see Table 1) neutralized BCV infection. Despite the observation that this was only 10% more than was observed for pre-pandemic sera (24%), the percentage of post-pandemic sera able to neutralize high concentrations of BCV was significantly higher than observed for pre-pandemic sera, i.e., a similar difference as observed between panels of human pre- and post-pandemic sera (Figure 4A). Moreover, in case percentages were calculated separately for the Peel and Veluwe regions, post-pandemic sera of the Peel region were largely responsible for this increase in the percentage of BCV-neutralizing sera and also for the higher percentage of sera able to neutralize a high concentration of BCV (Figure 4C).

### 3.5. Reactivity of Sera in SARS-CoV-2 NP and S ELISAs

**Figure 5:**
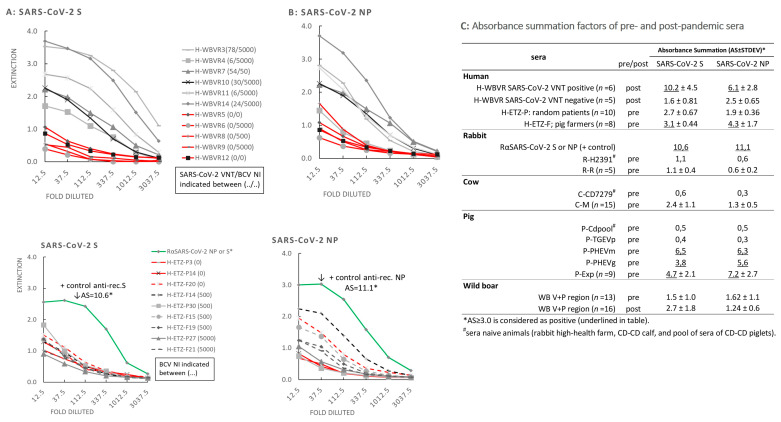
Reactivity of sera in SARS-CoV-2 S and NP ELISA. Reactivity of human pre- (H-ETZ-P and H-ETZ-F) and post-pandemic (H-WBVR) sera with coated recombinant proteins of SARS-CoV-2 S (**A**) and NP (**B**) in an indirect ELISA. BCV-NI’s and M96 SARS-CoV-2 VNT titers (only for post-pandemic sera) of tested sera are displayed between brackets in the legends of the graphs. Positive control sera prepared in rabbits (RαSARS-CoV-2 S or NP) are indicated with and asterisk [2] (**C**) Absorbance summation factor(s) measured for naïve animal sera, sera of piglets experimentally infected with PHEV and TGEV and panels of pre- and post-pandemic human, wild boar and farm animal sera. For panels of (n) sera the average “absorbance summation” factor (AS) [32] and STDEV were calculated. * An AS ≥ 3 was considered positive (see material and methods). Details about the origin of the sera are provided in Supplementary Table S1.

The WBVR panel of human sera (H-WBVR) with known SARS-CoV-2 VNT titer and BCV-NI was tested for binding to coated SARS-CoV-2 NP and S (S1–S2) recombinant proteins produced in insect cells in an indirect ELISA. All H-WBVR SARS-CoV-2 VNT-positive sera (SARS-CoV-2 convalescent) reacted with the rec. NP and S proteins of SARS-CoV-2 (Figure 5A,B, upper graphs), scoring a significantly higher AS factor than observed for SARS-CoV-2 VNT-negative sera. Sera from naive calves, rabbits and piglets and sera of piglets experimentally infected with the α-CoV TGEV did not cross-react with recombinant (rec.) S and NP of SARS-CoV-1/2 (AS scores are provided in the inserted table of Figure 5). Testing of a selection of human pre-pandemic sera (H-ETZ-P and H-ETZ-F) with known BCV-NI showed that only a few of the sera from the pig farmer panel (H-ETZ-F) weakly cross-reacted with rec. N and S of SARS-CoV-2 (Figure 5A,B, interrupted lines in bottom graphs). However, no correlation was found between AS scores and BCV-NIs for these human pre-pandemic sera and also not for the above-tested H-WBVR SARS-CoV-2-convalescent panel. Also, no cross-reactivity of BCV-neutralizing pre-pandemic sera of cows and rabbits was observed. In contrast, sera of pigs experimentally infected with PHEV, P-PHEVm and P-PHEVg cross-reacted with both S and NP of SARS-CoV-2 with AS scores of 6.5 and 6.3, respectively. Cross-reactivity of pig sera was also observed for seven out of nine pre-pandemic pig sera collected from a single farm in 2006 (P-Exp), scoring an average AS (*n* = 9) of 4.7 and 7.2 for S and NP, respectively. Interestingly, all these nine sera were able to neutralize the α-CoV TGEV, only two of these sera showed a weak neutralizing activity for BCV, and four of these sera stained PHEV (see Table 1).

### 3.6. BCV-Neutralizing Animal Sera Do Not Neutralize SARS-CoV-2 in a Standard M96 SARS-CoV-2 VNT Test

A selection of farm animal and wild boar sera with known NI for BCV were tested in the standard M96 SARS-CoV-2 VNT test [27]. Most of these sera scored a high BCV-NI of 5000 TCID_50_/well at a serum dilution of 1:50. None of the selected pig, cow and rabbit sera were able to neutralize 50 TCID_50_/M96 well of SARS-CoV-2 in case sera were serially diluted, starting with a dilution of 1:10 (Table 2). For five out of fourteen post-pandemic wild boar sera, a relatively weak neutralization activity towards SARS-CoV-2 was observed.

## 4. Discussion

Amino acid changes, small deletions and an insertion in the spike protein of SARS-CoV-2 VOCs are mainly restricted to the S1 subunit, including NTD, RBD and the S1/S2-furin cleavage site [34]. In comparison, only a few mutations were found in the S2 subunit of certain SARS-CoV-2 lineages (e.g., N856K in B.1.1.529) [35]. This implies that the S2 region is genetically stable and not subjected to immunological pressure of antibodies and/or activated T cells and does not tolerate many mutations in its functional domains. The S2′ peptide stretch that we used for antibody production in rabbits is located in the N-terminal half of S2. This conserved region in the S protein of CoV is a vital part of the tertiary structure of the S2′ cleavage site and contributes to the fusion-mediated entry of CoV into susceptible cells [29,36]. Our prepared IgG fraction produced from bleeds of immunized rabbits reacted in an ELISA with rec. SARS-CoV-2 S protein produced in insect cells and stained several α- and β-CoV-infected cells, including cells infected with SARS-CoV-2. However, it neutralized neither authentic SARS-CoV-2 nor pseudoviruses bearing wildtype or mutant SARS-CoV-2-S in vitro [29]. This, despite our 13 aa long peptide, was completely homologous to 13 amino acid residues within the 20 aa long FP linear cold-spot epitope that was shown to bind neutralizing antibodies present in sera of SARS-CoV-2 convalescent humans [12]. The shorter length of our peptide and/or a poor accessibility of this epitope in the 3D structure of the S protein trimer (e.g., due to shielding with glycan moieties attached to residues in the vicinity of the epitope) may weaken or prevent binding of our rabbit antibodies to in vitro produced CoV particles. Nevertheless, our data show that the anti-S2′ rabbit antibodies detect the FP epitope in cells infected with a broad range of CoV, confirming the genetic stability of the genomic region encoding this epitope. This makes our antibody preparation a ready-to-use reagent for the serologic detection of novel α- and β-CoV and SARS-CoV-2 VOCs that may spill over from the animal reservoir to humans, or vice versa, in the future [37]. 

The loss of infectivity of target virus in neutralization assays due to nonspecific binding of antibodies or due to high concentrations of (heterologous) serum components interfering with the infection process at the surface of susceptible cells may lead to false-positive VNT titers for sera [38,39]. In addition, the antibody-dependent enhancement (ADE) of virus infection in VNT assays due to the binding of sub- and non-neutralizing antibodies to virus particles may enhance viral infections [40]. We, therefore, developed neutralization assays for BCV, SARS-CoV-2 and TGEV using high ascending virus concentrations, a fixed serum dilution of 1:50 instead of serial dilutions and incubated virus–serum mixtures in a serum-free culture medium. Testing of sera collected from naïve animals, naïve animals experimentally infected or immunized with CoV and sera of SARS-CoV-2 of non- and convalescent humans showed that virus infection in these assays was not inhibited or enhanced by heterologous serum components at a dilution of 1:50. Moreover, we showed that our assays were able to detect cross-neutralizing antibodies in heterologous sera specifically directed against either a β-CoV or an α-CoV. Using this approach, we were able to partly characterize the antibody responses in humans, wild boar and farm animals elicited by past infections with CoV. 

The protein sequence of the S protein of BCV has 92% sequence identity to the S protein of HCoV-OC43 [23]. Several recent studies indicated that pre-pandemic infections with HCoV-OC43 or other related seasonal CoV strains were responsible for eliciting SARS-CoV-2 cross-reacting antibodies [7,8,41]. In immunological binding assays, it was shown that anti-OC43 and HKU1 IgG antibodies in pre-pandemic sera collected from children and adults cross-reacted with recombinant SARS-CoV-2 NP and S antigens [8]. In addition, it was shown that the concentration and/or affinity of anti-HCoV-OC43 S and -HKU1 S antibodies in sera from individuals who endured a COVID-19 infection were significantly higher compared to concentrations in pre-pandemic sera [7,8]. We also observed a similar increase in concentration/affinity of BCV-neutralizing antibodies and antibodies reacting with PHEV-infected cells. The percentage of SARS-CoV-2-convalescent sera able to neutralize BCV and stain PHEV was significantly higher (see Table 1). Also, a higher percentage of these convalescent sera was able to neutralize the highest concentrations of BCV we tested (5000 TCID_50_/well: see Figure 4A) and stain PHEV-infected cells at a higher serum dilution (Figure 4B). This is in agreement with the abovementioned serological studies with common-cold-associated HCoV [7,8] and confirms conclusions drawn by these studies that a memory antibody response directed against epitopes exposed on the surface of a broad range of HCoV (HCoV-OC43, HCoV-229E, HCoV-NL63, and HCoV-HKU1) was activated after exposure to SARS-CoV-2 [7,8,41]. Also, SARS-CoV-2-reactive CD4+ T cells were identified in 40%–60% of individuals that never encountered SARS-CoV-2 antigens [42]. It was suggested that this cross-reactive T-cell recognition of conserved CoV epitopes was one of the factors underlying the differences in the severity of COVID-19 disease observed in immune-competent healthy individuals [11,43]. Exposure to SARS-CoV-2 may trigger memory CD4+ T cells to initiate the selection and maturation of plasma cells and high-affinity antibody-producing memory B cells specific for these conserved epitopes in the germinal centers of the lymph nodes [44]. Therefore, the identification of these CoV-conserved epitopes and their cognate T cells may contribute to the development of vaccines that confer a broader and perhaps longer-lasting (cross)-protection against circulating SARS-CoV-2 variants and future emerging VOCs. Until now, animals like pigs and rabbits are typed as non-permissive hosts for existing SARS-CoV-2 variants [18,20]. Both pigs and rabbits can be obtained as naive (CD-CD) and seropositive for β-CoV (e.g., after infection with rabbit coronavirus HKU14 and porcine PHEV). This makes the blood of these animals an adequate and sufficient source of immune cells to study the mechanisms responsible for the activation of a T-cell-mediated memory response against conserved CoV-epitopes. Pigs and rabbits may serve as naturally primed model animals to test the potency of antigens or vaccines of CoV, including SARS-CoV-2, to activate such a memory antibody response.

Despite our group and others observing activation of an antibody memory response towards common CoV epitopes in individuals that endured a SARS-CoV-2 infection, we did not find a positive correlation between the BCV-NI or PHEV staining scores and SARS-CoV-2 VNT titers in convalescent sera (see Supplementary Figure S4A,B). In the past 3 years, sets of pre-pandemic human sera have been studied extensively in SARS-CoV-2 neutralization tests. These tests were mainly conducted with surrogate SARS-CoV-2 viruses and recombinant viruses pseudo-typed with the spike protein of SARS-CoV-2, and mostly focused on binding of antibodies to the S protein and its RBD. However, to our knowledge, none of these serological studies showed that pre-pandemic sera were able to effectively neutralize native SARS-CoV-2 isolates in vitro [45,46]. Furthermore, this was only limited to no cross-binding/-neutralization of SARS-CoV-1 and MERS-CoV observed by antibodies in the plasma of COVID-19 patients with neutralizing activity towards SARS-CoV-2 [45,46]. Binding of the RBD to ACE2 receptor imposes conformational changes in the SARS-CoV-2 spike trimer [1]. This could result in dissociation of these antibodies from the SARS-CoV-2 virus particles. In line with this, none of the pre-pandemic human sera with high neutralizing activity for BCV showed considerable reactivity for rec. SARS-CoV-2 S in the ELISA, whereas all tested convalescent human sera with a SARS-CoV-2 VNT titer did (Figure 5A,C), even when their SARS-CoV-2 VNT titer was relatively low (Figure 5A). In addition, epitopes on the surface proteins S, M and/or E located beneath the protruding top of the spikes (i.e., closer to, or on the core surface) may be unreachable for these cross-reactive antibodies due to steric hindrance by the globular head of the SARS-CoV-2 spikes and/or the glycosyl groups attached to the S proteins [47]. 

Testing of pre-pandemic sera from sheep, pigs and rabbits suggested that cross-neutralization of BCV was restricted to sera collected from specific herds or farms (see Table 1). BCV neutralization was observed for all 16 pre-pandemic cow sera collected from three independent dairy farms, reflecting the endemic state of BCV in Central Europe [21]. As observed for humans, past infections with (species)-specific β-CoV (e.g., rabbit coronavirus HKU14 and PHEV) or other circulating yet uncharacterized CoV (or variants) in animals elicited these cross-reactive antibodies. Similar to that observed for pre-pandemic human sera, pre-pandemic sera of rabbits and cows able to neutralize BCV and stain PHEV-infected cells were not able to neutralize SARS-CoV-2 in a standard (M96) neutralization assay and did not react with rec. SARS-CoV-2 S and N proteins in an ELISA. Interestingly, seven out of nine sera collected from juvenile pigs in a farm in 2006 (P-Exp) all reacted positively with rec. S and NP in ELISAs. Furthermore, all nine pig sera effectively neutralized TGEV, but only two of these nine sera neutralized BCV. This effective neutralization of TGEV suggests that past infection(s) with a TGEV-like α-CoV virus was responsible for eliciting these (cross)-reacting antibodies. A possible candidate for this could be “Porcine respiratory coronavirus” (PRCV). PRCV evolved from TGEV by in vivo selection of variants carrying mutations and deletions in their RNA genome in the 1980s. The emergence of PRCV expelled TGEV infections from the pig population in the 1990s, and PRCV became endemic in pigs thereafter [22,48]. This transition also resulted in a change in tissue tropism, i.e., from a severe enteric virus (TGEV) to a mild respiratory viral pathogen (PRCV) [24]. The cross-reactivity of these pre-pandemic P-Exp sera in the ELISAs for SARS-CoV-1/2 N and S (see inserted table in Figure 5) suggested that such a TGEV-like α-CoV may also share common epitopes with SARS-CoV-2. Because numerous pre-pandemic sera collected randomly from finishing pigs at a slaughterhouse (P-F Table 1) in 2007 also stained PHEV and effectively neutralized BCV in addition to neutralization of TGEV, it is likely that, in addition to past infections with an α -CoV-like PRCV, a β-CoV in the Dutch pig population accounted for this broad cross-reactivity for CoV. The β-CoV PHEV could be a candidate for this. Remarkedly, the percentage of pre-pandemic sera collected from pig farmers, able to neutralize BCV infection and stain PHEV-infected cells, was significantly higher compared to pre-pandemic sera of random patients (compare pre-pandemic H-ETZ-F and H-ETZ-P panels in Table 1). Frequent exposure of pig farmers to CoV circulating among their pigs (e.g., PHEV or another unknown β-CoV) may have elicited an antibody response towards conserved β-CoV epitopes and kept it sustained. Larger panels of serum samples collected from individuals working intensively with pigs must be tested to find supporting evidence for this hypothesis. 

Similar to that observed for human sera, we also observed an increased percentage of post-pandemic sera collected from wild boars in the urban “Peel” region able to neutralize high concentrations of BCV. Unfortunately, only serum samples were available from these hunted wild boars. Diagnosis of SARS-CoV-2 infection by PCR analysis of serum samples is not always reliable and scores low detection rates [49], and references herein. We tested 40 randomly selected post-pandemic wild boar sera from the Veluwe and Peel regions by PCR and did not detect SARS-CoV-2 E-gene genomic fragments in these sera. Also, no SARS-CoV-2 PCR positives were detected in a panel of 422 fecal samples collected from free-living wild boars in Croatia during the second COVID-19 wave [50]. This suggests that virus replication of SARS-CoV-2 in wild boars is either inefficient or does not occur at all. However, this does not rule out the possibility that the increase in number and strength of BCV neutralization observed for post-pandemic wild boar sera from the Peel region was not caused by exposure of the wild boars to SARS-CoV-2. Similar to that observed in other non-naïve animals [15], low-level replication of SARS-CoV-2 may not be detected and/or infection with SARS-CoV-2 may be symptomless. Because the wild boars were hunted during the 2nd–4th COVID-19 waves in The Netherlands, human waste or excreta (e.g., in sewage, surface water or food remains) in the urbanized Peel region may account for the exposure of SARS-CoV-2 to these free-living omnivores. This exposure may have triggered a memory antibody response, e.g., after cross-reactive T-cell recognition of common or structurally related epitopes exposed on the surface of both SARS-CoV-2 and a CoV that circulated in the wild boar population [42]. Further research is needed to find additional evidence for whether environmental exposure to a β-CoV like SARS-CoV-2 is sufficient to activate such a “memory” antibody response.

## 5. Conclusions

In summary, cross-reaction of pre- and post-pandemic panels of human and animal sera with BCV and PHEV confirmed that past infection(s) with β-CoV elicited antibodies directed against epitopes exposed on the surface of a broad range of heterologous CoV, including SARS-CoV-2 and β-CoV circulating in farm animals. Despite particular heterologous sera recognizing epitopes on immobilized rec. SARS-CoV-2 S and NP proteins, they failed to neutralize SARS-CoV-2 infection in vitro. This emphasizes the unique surface structure SARS-CoV-2 acquired during the (proposed) multiple zoonotic spill-over events before it emerged in humans [51]. Further characterization of these conserved epitopes and the mechanisms responsible for eliciting a memory antibody response directed against these epitopes may contribute to the development of vaccines that provide an acceptable level of (cross)-protection against future arising SARS-CoV-2 VOCs or other emerging “threatful” coronaviruses that could spill over from animals to humans and vice versa in the future. In general, the development of such vaccines requires extensive research, testing and regulatory approval processes to ensure their safety and efficacy. Vaccines that target conserved epitopes can enhance the efficacy of vaccines and improve vaccination strategies and, thereby, the preparedness for potential future outbreaks. Especially in the case of SARS-CoV-2, for which future arising VOCs may escape immunity, developed better targeted vaccines may be protective for a longer period at the population level or evoke longer-lasting immunity in susceptible individuals. The cost to develop such vaccines and of measures to prevent future zoonotic spill overs would be negligible to the total costs of a worldwide future pandemic [52]. 

## Figures and Tables

**Table 1:** Human, wild boar and farm animal sera with cross-reactivity for BCV, PHEV and TGEV.

Species and Origin Field Sera ^&^	Year Collected (Pre- or Post-Pandemic)	BCV Neutralization Index (NI)					TGEV Neutralization Index (NI)					PHEV Immune Staining			
		Number Tested	Number Positive (%) ^$^	5000	500	50	Number Tested	Number Positive (%) ^$^	10,000	1000	100	Number Tested (BCV+)	Number Positive (%) ^$^	1:750 ** [++]	1:250 ** [+]
Goat															
G (2 farms NL *)	2012 (pre)	20	0	0	0	0	NT	−	−	−	−	NT	−	−	−
Rabbit															
R-R (regular husbandry 5–7 wks)	2019-21 (pre-post)	20	12 (60%)	4	1	7	5 (5)	0	0	0	0	4 (4)	3	2	1
Cow															
C-M (dairy cows 3 farms NL)	2010 (pre)	16	16 (100%)	6	5	5	9 (9)	0	0	0	0	5 (4)	2	1	1
Sheep															
S-H herd 1 (NL)	2012 (pre)	4	4	1	2	1	4 (4)	0	0	0	0	4 (4)	0	0	0
S-H herd 2 (NL)	2012 (pre)	2	0	0	0	0	NT	−	−	−	−	2 (0)	0	0	0
S-H herd 3 (NL)	2012 (pre)	5	0	0	0	0	NT	−	−	−	−	NT	−	−	−
S-SPF (SPF herd France)	2012 (pre)	9	0	0	0	0	NT	−	−	−	−	NT	−	−	−
Pig															
P-F (finishing pigs NL)	2007 (pre)	42	19 (45%)	2	12	5	37 (18)	21 (57%)	7	9	5	40 (17)	11 (28%)	2	9
P-Exp (10–12 wks old piglets NL)	2006 (pre)	9	2	0	1	1	9 (2 )	9 (100%)	1	5	3	9 (2)	4	1	3
P-S ([pregnant-]sows farm NL)	2021 (post)	6	1	0	0	1	6 (1)	3	2	1	0	2 (1)	0	0	0
P-P (weaned piglets farm NL)	2021 (post)	21	0 (0%)	0	0	0	21 (0)	10 (48%)	1	2	7	3 (0)	0	0	0
P-FA (fattening pigs 2 farms NL)	2021 (post)	56	18 (32%)	7	6	5	28 (18)	19 (68%)	2	11	6	NT	−	−	−
Wild Boar (all ages)															
WB-V and WB-P (Veluwe and Peel area NL)	2018 (pre)	91	22 (24%)	6	5	11	53 (16)	2 (3.8%)	0	2	0	19 (6)	2 (11%)	1	1
WB-V and WB-P (Veluwe and Peel area NL)	2021-22 (post)	185	63 (34%)	31	20	12	75 (59)	4 (5.3%)	1	2	1	16 (16)	9 (56%)	2	7
Human															
H-ETZ-F (pig farmers)	2018 (pre)	22	15 (68%)	1	4	10	12 (8)	0 (0%)	0	0	0	15 (15)	8 (53%)	0	8
H-ETZ-P (random patients)	2018 (pre)	30	11 (37%)	4	2	5	12 (6)	0 (0%)	0	0	0	11 (11)	4 (36%)	0	4
H-ETZ-IC, H-ETZ-W and H-WBVR (SARS-CoV-2 PCR+)	2020 (post)	78	61 (78%)	36	14	11	14 (9 )	0 (0%)	0	0	0	78 (61)	60 (77%)	38	22

^&^ all sera were tested in BCV and TGEV neutralization assays in an 1:50 dilution with 3 ascending concentrations of virus; ^$^ percentage positive sera in neutralization tests and PHEV staining are listed for panels that contain at least 10 sera; * NL = the Netherlands; ** 1:750 [++] or 1:250 [+]: serum dilution staining PHEV-infected cells.

**Table 2:** SARS-CoV-2 VNT titers of BCV-neutralizing wild boar and farm animal sera.

Species and Origin Field Sera ^&^	Pre- or Post-Pandemic	Number (*n*) of Sera	BCV NI * (1:50 Dilution)	SARS-CoV-2 VNT Titer **^$^ (M96-Test)
Hamster				
HAM-d0 (pre-serum before infection)	(−) control	1	0	<10
HAM-d21 (SARS-CoV-2 infected)	(+) control	1	0	250
Rabbit				
R-R (regular husbandry 5–7 wks)	pre-	2	50|5000	<10 (*n* = 2)
Cow				
C-M (dairy cows NL)	pre-	11	5000 (*n* = 11)	<10 (*n* = 11)
Pig				
P-F (finishing pigs NL)	pre-	2	500|5000	<10 (*n* = 2)
P-Exp (10–12 wks old piglets NL)	pre-	1	0	<10 (*n* = 1)
P-S ([pregnant-]sows farm NL)	post	1	50	<10 (*n* = 1)
Wild Boar				
WB-V (Veluwe region NL)	post	6	5000 (*n* = 6)	<10 (*n* = 3)|10, 30, 50 (*n* = 3)
WB-P (Peel region NL)	post	8	500 (*n* = 1)|5000 (*n* = 7)	<10 (*n* = 6)|10, 15 (*n* = 2)

^&^ For details about the origin of the sera, see Supplementary Table S1; * 500 (*n* = 1)|5000 (*n* = 7): BCV NI = 500 for 1 serum sample and 5000 for 7 samples; $ VNT titer: dilution of serum neutralizing 50 TCID50/well of SARS-CoV-2 for 90%. Dilutions of <1:10 were considered as negative (<10); ** <10 (*n* = 3)|10, 30, 50 (*n* = 3): SARS-CoV-2 VNT titer < 10 for 3 serum samples |VNT titers of 3 positive serum samples are 20, 30 and 50.

## Data Availability

Data are contained within the article and Supplementary Materials.

## Supplementary Materials

The following supporting information can be downloaded at: https://www.mdpi.com/article/10.3390/v16010034/s1. A pdf file with Supplementary Figures S1–S5 and Supplementary Tables S1 and S2. Supplementary Figure S1: Effect of anti-S2-WBVR3 IgG antibodies on infection process (% Entry) of vesicular stomatitis virus (VSV) pseudo-typed with WT and variants of the spike protein of SARS-CoV-2. Supplementary Table S1.; Origin of human and animal sera tested in this study. Supplementary Figure S2: Validation of TGEV neutralization assay with sera from (experimentally) infected animals and human lab workers WBVR. Supplementary Figure S3: Immune staining of PHEV-infected SK6 cells. Supplementary Figure S4A: Chi-square statistic contingency tables comparing number of sera per BCV-NI category and per PHEV-staining category for pre- and post-pandemic panels of human and wild boar sera. Supplementary Figure S4B: SARS-CoV-2 VNT titer versus BCV-NI and versus staining of PHEV-infected SK6 cells. Supplementary Figure S5: Map of Veluwe and Peel regions in The Netherlands with locations of hunted wild boars. Supplementary Table S2: Species-specific HRP-conjugates used as secondary antibodies for immune-staining of virus-infected cells and in ELISA’s.
