# Supplementary material for: Cross-Reactivity of Human, Wild Boar, and Farm Animal Sera from Pre- and Post-Pandemic Periods with Alpha- and Βeta-Coronaviruses (CoV), including SARS-CoV-2
Source: Viruses. 2023 Dec 23;16(1):34. doi: 10.3390/v16010034 (PMC10821012; doi:10.3390/v16010034)
Supplement: Supplementary file 1 [file viruses-16-00034-s001.zip › viruses-2763142-supplementary.pdf]

Supplementary Figure S1

Effect of anti-S2-WBVR3 IgG antibodies on infection process (% Entry) of vesicular stomatitis virus (VSV) pseudo-typed with WT and variants of the spike protein of SARS-CoV-2.

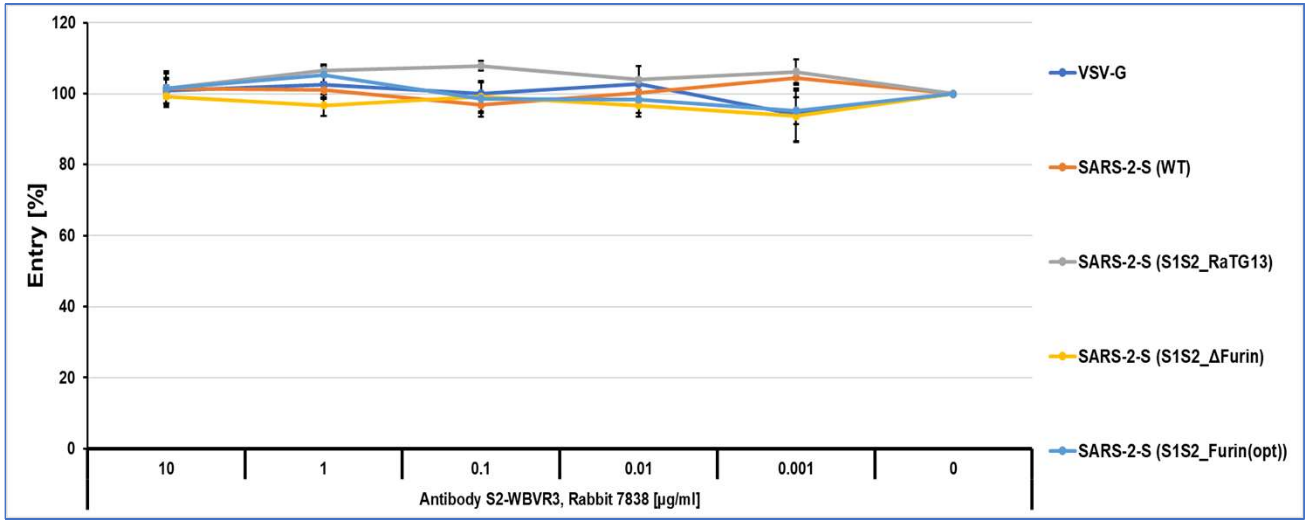

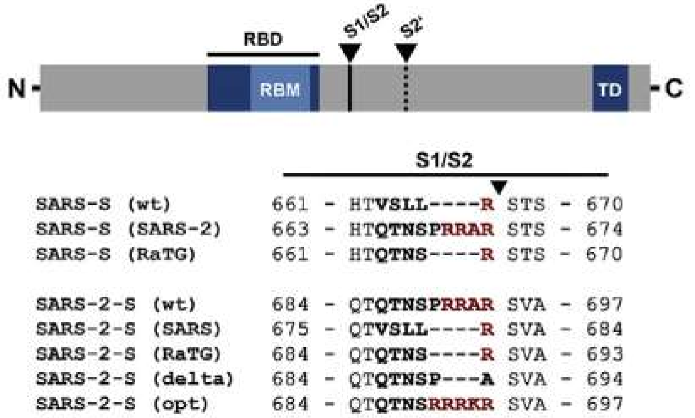

SARS-S and SARS-2-S S1/S2 mutants analyzed (for detailed information see Fig. 2A. in "A Multibasic Cleavage Site in the Spike Protein of SARS-CoV-2 Is Essential for Infection of Human Lung Cells": Hoffmann et. al. Mol Cell. 2020;78(4):779-784.e5(reference 27).

Experiments conducted by Dr. Markus Hoffmann's lab. German Primate Center, Infection Biology Unit, Göttingen.

Supplementary Table S1. Origin of human and animal sera tested in this study

species	age	year	collected before (pre) or after start pandemic (post)	# sera	notation used in tables and figures	source
**field sera**						
**Human**	adult	2018	pre	30	H-ETZ-P#	random patients ETZ hospital NL
	adult	2018	pre	22	H-ETZ-F#	pig farmers North Brabant region NL
	adult	2020	post	5	H-WBVR#	lab workers WBVR NL, PCR-negative for SARS-CoV-2
	adult	2020	post	6	H-WBVR#	lab workers WBVR NL, PCR-positive for SARS-CoV-2
	adult	2020-2021	post	12	H-ETZ-IC#	IC patients ETZ hospital NL, PCR positive for SARS-CoV-2 (autumn and winter 2020-2021)
	adult	2020	post	60	H-ETZ-W#	hospital workers ETZ hospital NL, PCR positive for SARS-CoV-2 (April and May 2020)
**Rabbit**	5-7 wks	2019	pre	4	R-H#	pre-serum laboratory rabbits "high health husbandry"
	5-7 wks	2019-2020	pre	20	R-R#	pre-sera laboratory rabbits "regular husbandry"
**Pig**	adult	2007	pre	20	P-F#	finishing pigs regular slaugtherhouse NL
	10-12 wks	2006	pre	9	P-Exp#	regular farm NL (used in a Hepatitis E virus transmission experiment)
	6-7 wks	2021	post	21	P-P#	weaned piglets farm NL (same farm as P-S)
	adult	2021	post	6	P-S#	(pregnant-) sows pig farm NL (same farm as P-P)
	12-20 wks	2021	post	56	P-FA#	fattening pigs 2 regular farms of same owner NL (28 of each farm)
**Wild boar**	unknown	2018	pre	43	WB-V#	sera hunted wild boar collected in Veluwe area NL (2018 week 1 through week 52)
	unknown	2018	pre	47	WB-P#	sera hunted wild boar in Peel area NL (2018 week 1 through week 52)
	unknown	2021-2022	post	64	WB-V#	sera hunted wild boar in Veluwe area NL (januari 2021 until march 2022)
	unknown	2021-2022	post	114	WB-P#	sera hunted wild boar in Peel area NL (januari 2021 until march 2022)
**Cow**	adult	2010	pre	16	C-M#	milk cows of 3 farms NL
**Sheep**	adult	2012	pre	11	S-H#	2, 4 and 5 from 3 different local herds NL
	adult	2012	pre	10	S-SPF#	SPF herd INRA France
**Goat**	adult	2019	pre	20	G#	2 different farms NL (10 of each farm)
**experimental sera**						
**Rabbit**	20 wks	2020	NA	1	αS2’-IgG	affinity-purified rabbit IgG's directed against S2 peptide WBVR3
	20 wks	2020	NA	1	RαSARS-CoV-2 NP (+C)	positive control serum for SARS-CoV 1&2 NP ELISA (see reference 27)
	20 wks	2020	NA	1	RαSARS-CoV-2 S (+C)	positive control serum for SARS-CoV 1&2 S ELISA (see reference 27)
**Cow**	1d	2021	NA	4	C-CD#	CD-CD calfs WBVR
**Syrian hamster**	7 wks	2020	NA	1	HAM-d0	pre-serum hamster before infection with SARS-CoV-2 (0 d) (see refrence 27)
	10 wks	2020	NA	1	HAM-d21	hamster experimentally infected with SARS-CoV-2 (21 d.p.i.) (see refrence 27)
**Pig**	adult	2006	NA	1	P-PHEVg	PHEV immunized pig (gift Univ. of Gent Belgium)
	adult	unknown	pre	1	P-PHEVm	PHEV immunized pig (gift Univ. of Minnesota USA)
	piglet	1989	NA	1	P-PRCTGEVm	experimental infection with PRCV/TGEV strain Miller
	piglet	1988	NA	1	P-TGEVp	experimental infection with TGEV strain Purdue
	piglet	1988	NA	1	P-PEDV	experimental infection with PEDV strain CV777
	1d	2019	NA	1	P-CDpool	pooled sera of CD-CD piglets WBVR

Supplementary Figure S2: Validation of TGEV neutralization assay with sera from (experimentally) infected animals and human lab workers WBVR.

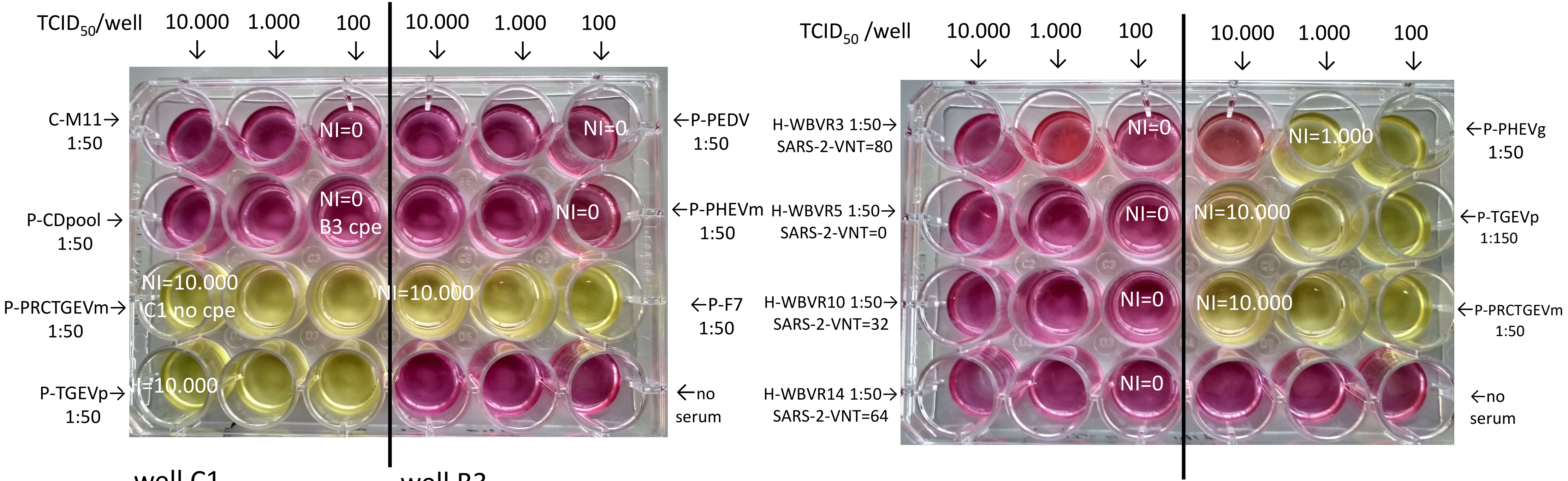

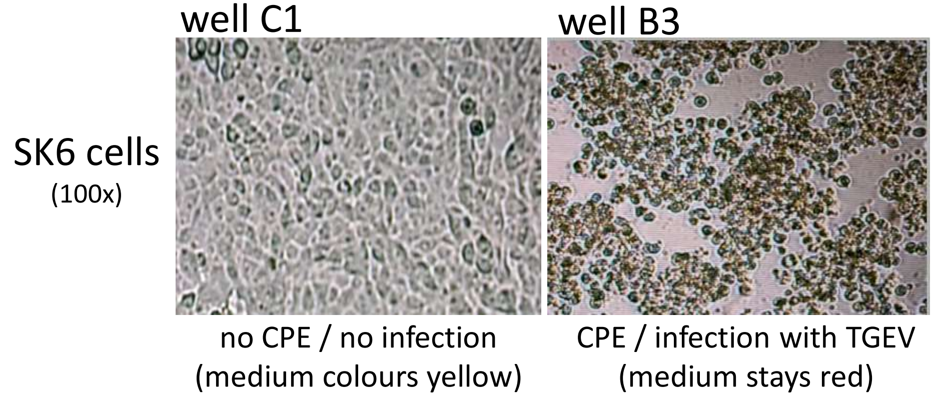

notation	origin serum (year collected)	NI at 1:50 dilution
P-PHEVm	pig immunized with PHEV (pre-pandemic: gift Univ. of Minnesota)	0
P-PHEVg	PHEV immunized pig (2006: gift Univ. of Gent Belgium)	1.000
P-PEDV	piglet experimentally infected with PEDV strain CV777 (1988)	0
P-TGEVp	piglet experimentally infected with TGEV strain Purdue (1988)	10.000
P-PRCTGEVm	piglet experimentally infected with PRCV/TGEV strain Miller (1989)	10.000
C-M11	cow of a dairy farm Netherlands, positive for anti-BCV antibodies (2016)	0
H-WBVR#	WBVR3, 5, 10, and 14, M96 SARS-CoV-2 VNT titers indicated in figure (2020)	0/0/0/0
P-F7	finishing pig regular slaughterhouse NL (2007)	10.000
P-CDpool	pooled sera of CD-CD piglets WBVR (2019)	0

Supplementary Figure S3: Immune staining of PHEV infected SK6 cells **A** : Staining of PHEV infected and mock infected SK6 cells

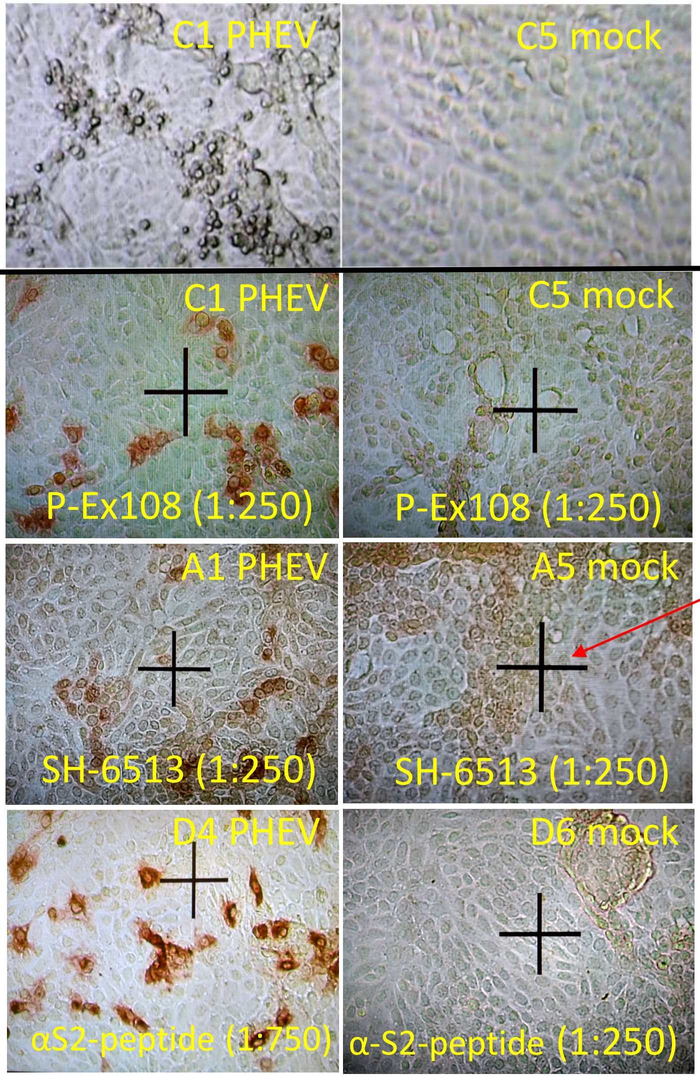

before staining

-left picture: CPE induced by PHEV 4 dpi. (appearance of clusters of apoptotic cells)

-right picture: mock infected cells.

Stained

+ marks the centre of a well (no indication for positive staining!)

B : Detection of positive and negative PHEV staining based on detected number of infected foci per 2 cm² well (blue counts) using the AID vSpot imaging apparatus (AID Autoimmun Diagnostika GmbH, Strassberg, Germany).

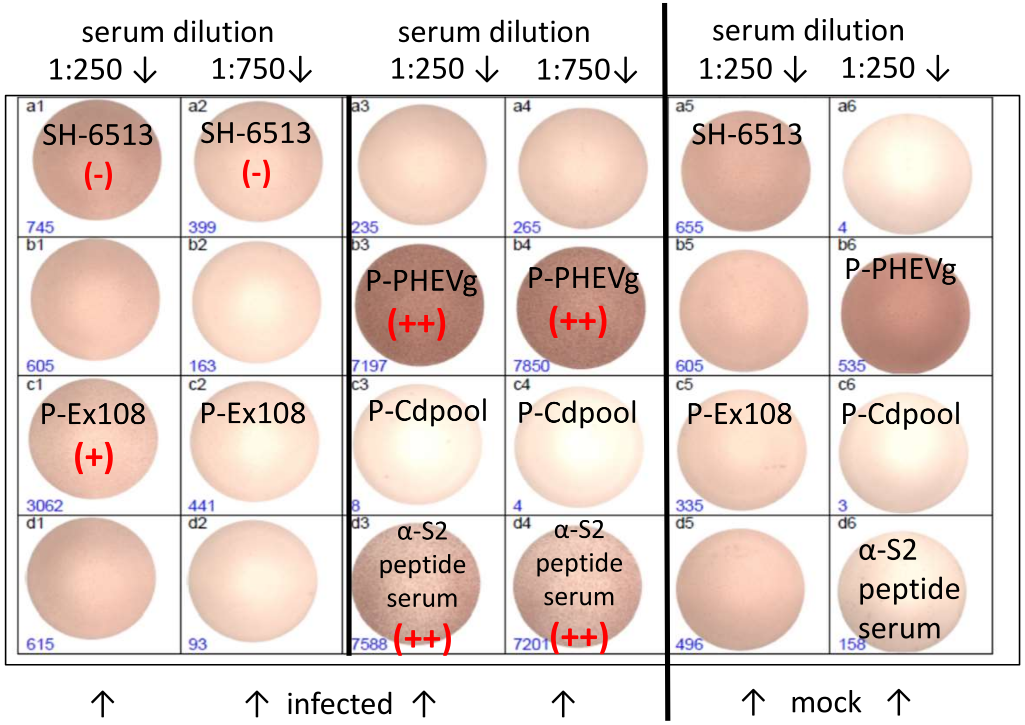

serum	# foci infected 1:250	# foci infected 1:750	# foci mock 1:250	PN value 1:250* (# infected/mock)	PN value 1:750* (# infected/mock)	PN>5 at dilution$	notation&
SH-6513	745	399	655	1.1	0.6	No	(-)
P-Exp108	3062	441	335	9.1	1.3	1:250	+
α-S2-peptide	7588	7201	158	48.0	45.6	1:750	++
P-PHEVg	7179	7850	605	11.9	13.0	1:750	++
P-Cdpool	8	4	3	2.7	1.3	No	(-)

*PN value: number of foci detected in infected wells at dilution of 1:250 or 1:750 divided by the number of foci in mock infected cells at an dilution of 1:250

$ serum dilution scoring a PN value >5

& scores: (++) 1:750 dilution of serum scoring a PN value >5, (+) 1:250 dilution scoring a PN value >5 and 1:750 does not, (-) PN<5 at serum dilution of 1:250.

Supplementary Figure S4A: Chi-square statistics of BCV-NI and staining of PHEV infected SK6 cells of data displayed in Figure S4.

Statistical analysis

Fig 4A Chi-square statistics

Results chi-square statistics of BCV neutralisation by pre-and post-pandemic human sera

category →	BCV NI=0	BCV NI=50	BCV NI=500	BCV NI=5000	total # sera
serum panels					
H-ETZ + WBVR SARS-CoV-2 convalescent	17 (25.80) [3.00]	11 (15.60) [1.36]	14 (12.00) [0.33]	36 (24.60) [5.28]	78
H-ETZ pre-pandemic	26 (17.20) [4.50]	15 (10.40) [2.03]	6 (8.00) [0.50]	5 (16.40) [7.92]	52
# sera per category →	43	26	20	41	130 (Grand Total)

DF=3, Critical X^2=7.82 at an alpha of 5%

The chi-square statistic is 24.9356. The p-value is .000016.The result is significant at p < .05.

The contingency table above provides the following information: the observed number of sera per BCV-NI category, (the expected cell totals) and [the chi-square statistic for each cell].

Results chi-square statistics BCV neutralisation by pre-and post-pandemic wild boar sera

category →	BCV NI=0	BCV NI=50	BCV NI=500	BCV NI=5000	total # sera
serum panels					
WB post-pandemic	122 (128.03) [0.28]	12 (15.42) [0.76]	20 (16.76) [0.63]	31 (24.80) [1.55]	185
WB pre-pandemic	69 (62.97) [0.58]	11 (7.58) [1.54]	5 (8.24) [1.28]	6 (12.20) [3.15]	91
# sera per category →	191	23	25	37	276 (Grand Total)

DF=3, Critical X^2=7.82 at an alpha of 5%

The chi-square statistic is 9.7598. The p-value is .020723.The result is significant at p < .05.

The contingency table above provides the following information: the observed number of sera per BCV-NI category, (the expected cell totals) and [the chi-square statistic for each cell].

Fig 4B Chi-square statistics

Results chi-square statistics of PHEV staining by pre-and post-pandemic human sera

category →	no staining [-]	staining at [1:250]	staining at [1:750]	total # sera
serum panels				
H-ETZ + WBVR SARS-CoV-2 convalescent	18 (24.00) [1.50]	22 (24.75) [0.31]	38 (29.25) [2.62]	78
H-ETZ pre-pandemic	14 (8.00) [4.50]	11 (8.25) [0.92]	1 (9.75) [7.85]	26
# sera per category →	32	33	39	104 (Grand Total)

DF=2, Critical X^2=5.99 at an alpha of 5%

The chi-square statistic is 17.6923. The p-value is .000144. The result is significant at p < .05.

The contingency table above provides the following information: the observed number of sera per PHEV-staining category, (the expected cell totals) and [the chi-square statistic for each cell].

Results chi-square statistics of PHEV staining by pre-and post-pandemic wild boar sera

category →	no staining [-]	staining at [1:250]	staining at [1:750]	total # sera
serum panels				
WB post-pandemic	16 (12.49) [0.99]	1 (4.34) [2.57]	2 (2.17) [0.01]	19
WB pre-pandemic	7 (10.51) [1.17]	7 (3.66) [3.06]	2 (1.83) [0.02]	16
# sera per category →	23	8	4	35 (Grand Total)

DF=2, Critical X^2=5.99 at an alpha of 5%

The chi-square statistic is 7.8221. The p-value is .02002. The result is significant at p < .05.

The contingency table above provides the following information: the observed number of sera per PHEV-staining category, (the expected cell totals) and [the chi-square statistic for each cell].

Fig 4C Chi-square statistics

Results chi-square statistics of BCV neutralisation by post-pandemic wild boar sera from two different locations in the Netherland

category →	BCV NI=0	BCV NI=50	BCV NI=500	BCV NI=5000	total # sera
post-pandemic Wild boar serum panels					
WB Peel rgion	65 (74.29) [1.16]	10 (7.69) [0.70]	14 (12.17) [0.28]	25 (19.85) [1.33]	114
WB Veluwe region	51 (41.71) [2.07]	2 (4.31) [1.24]	5 (6.83) [0.49]	6 (11.15) [2.38]	64
# sera per category →	116	12	19	31	178 (Grand Total)

DF=3, Critical X^2=7.82 at an alpha of 5%

The chi-square statistic is 9.6476. The p-value is .021812. The result is significant at p < .05.

The contingency table above provides the following information: the observed number of sera per BCV-NI category, (the expected cell totals) and [the chi-square statistic for each cell].

Supplementary Figure S4B: SARS-CoV-2 VNT titer versus BCV-NI (A) and staining of PHEV infected SK6 cells (B).

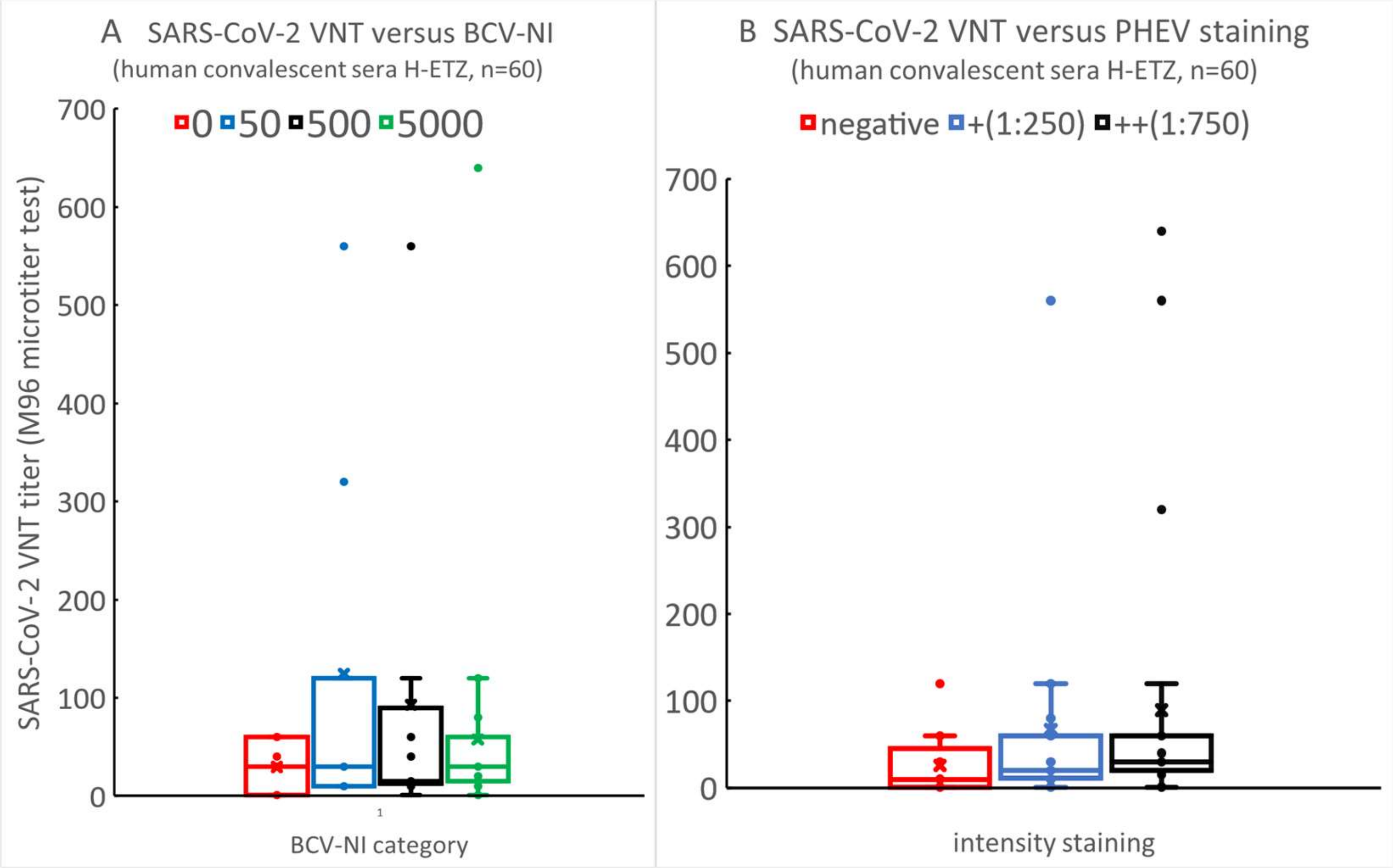

Supplementary Figure S5: Map of Veluwe and Peel regions in the Netherlands (locations of hunted wild boars).

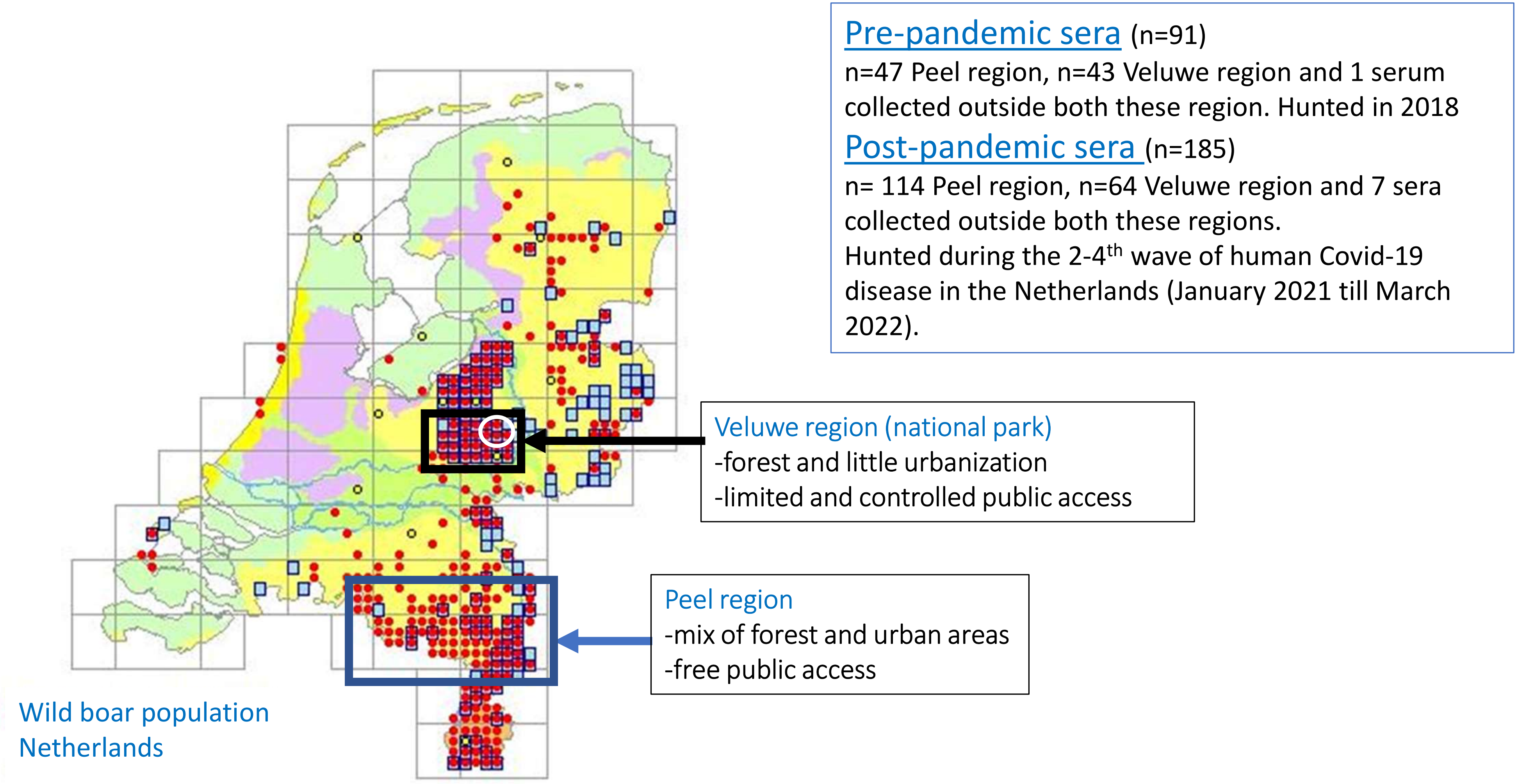

Supplementary Table S2. Species-specific HRP-conjugates used as secondary antibodies for immune-staining of virus-infected cells and ELISA's .

species	HRP conjugate	reference - firm - location
cow	Rabbit anti-Bovine IgG (H+L) Secondary Antibody, HRP	Thermo-fischer scientific - Bleiswijk, The Netherlands
hamster	Rabbit anti-Syrian Hamster IgG (H+L) Secondary Antibody, HRP	Thermo-fischer scientific - Bleiswijk, The Netherlands
human	Goat-anti-Human-IgG-IgM-IgA-H-L-Secondary-Antibody-Polyclonal HRP	Thermo-fischer scientific - Bleiswijk, The Netherlands
pig	Monoclonal antibody Anti-Swine Ig-L 27.2.1 WBVR	reference 31
rabbit	Goat Anti-Rabbit Immunoglobulins/HRP” (Dako)	Agilent - Abcoude the Netherlands
wild boar	Monoclonal antibody Anti-Swine Ig-L 27.2.1 WBVR	reference 31
sheep	Rabbit anti-Sheep IgG (H+L) Secondary Antibody, HRP	Thermo-fischer scientific - Bleiswijk, The Netherlands
